# Iron Nitride Nanoparticles for Enhanced Reductive Dechlorination of Trichloroethylene

**DOI:** 10.1021/acs.est.1c08282

**Published:** 2022-03-09

**Authors:** Miroslav Brumovský, Jana Oborná, Vesna Micić, Ondřej Malina, Josef Kašlík, Daniel Tunega, Miroslav Kolos, Thilo Hofmann, František Karlický, Jan Filip

**Affiliations:** † Department of Environmental Geosciences (EDGE), Centre for Microbiology and Environmental Systems Science, University of Vienna, Althanstrasse 14, UZA II, 1090 Vienna, Austria; ‡ Regional Centre of Advanced Technologies and Materials, Czech Advanced Technology and Research Institute (CATRIN), Palacký University Olomouc, Šlechtitelů 27, 779 00 Olomouc, Czech Republic; § Department of Forest- and Soil Sciences, Institute of Soil Research, University of Natural Resources and Life Sciences, Vienna, Peter-Jordan-Straße 82, 1190 Vienna, Austria; ∥ School of Pharmaceutical Science and Technology, Tianjin University, 300072 Tianjin, P.R. China; ⊥ Department of Physics, Faculty of Science, University of Ostrava, 701 03 Ostrava, Czech Republic

**Keywords:** Iron nitride, Nanoparticles, Zerovalent iron, Trichloroethylene, Dechlorination, Selectivity, Molecular modeling, Groundwater remediation

## Abstract

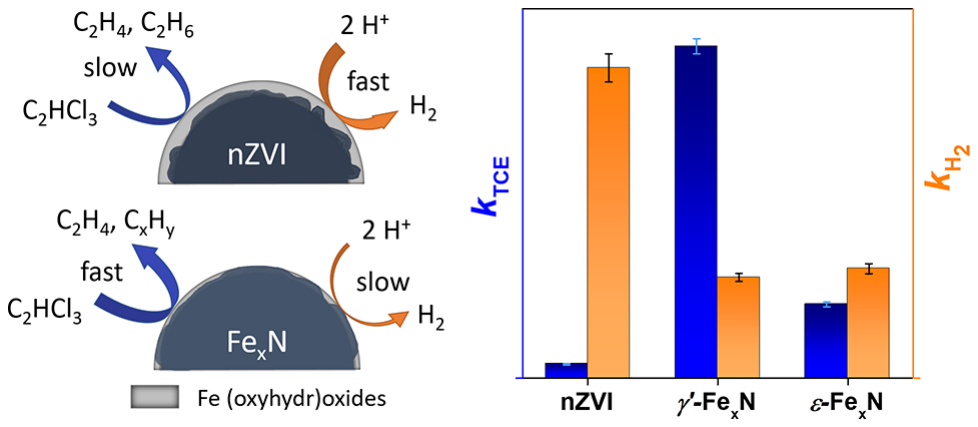

Nitriding has been used for decades to improve the corrosion resistance of iron and steel materials. Moreover, iron nitrides (Fe_*x*_N) have been shown to give an outstanding catalytic performance in a wide range of applications. We demonstrate that nitriding also substantially enhances the reactivity of zerovalent iron nanoparticles (nZVI) used for groundwater remediation, alongside reducing particle corrosion. Two different types of Fe_*x*_N nanoparticles were synthesized by passing gaseous NH_3_/N_2_ mixtures over pristine nZVI at elevated temperatures. The resulting particles were composed mostly of face-centered cubic (*γ′*-Fe_4_N) and hexagonal close-packed (*ε*-Fe_2–3_N) arrangements. Nitriding was found to increase the particles’ water contact angle and surface availability of iron in reduced forms. The two types of Fe_*x*_N nanoparticles showed a 20- and 5-fold increase in the trichloroethylene (TCE) dechlorination rate, compared to pristine nZVI, and about a 3-fold reduction in the hydrogen evolution rate. This was related to a low energy barrier of 27.0 kJ mol^–1^ for the first dechlorination step of TCE on the *γ′*-Fe_4_N(001) surface, as revealed by density functional theory calculations with an implicit solvation model. TCE dechlorination experiments with aged particles showed that the *γ′*-Fe_4_N nanoparticles retained high reactivity even after three months of aging. This combined theoretical-experimental study shows that Fe_*x*_N nanoparticles represent a new and potentially important tool for TCE dechlorination.

## Abstract 2

The nitriding of zerovalent iron nanoparticles leads to a substantial improvement in their reactivity and selectivity for trichloroethylene. The concurrent inhibition of iron corrosion enables better long-term performance.

## 1 Introduction

Chlorinated solvents (CSs) are one of the most frequent soil and groundwater contaminants worldwide.^1,2^ Due to their high density and high affinity for sorption, the remediation of CS-contaminated sites is especially demanding and costly.^2,3^ The injection of nanoscale zerovalent iron (nZVI) particles into contaminated aquifers has been proposed as a promising strategy for the in situ remediation of CSs.^4−6^ Remedial efforts employing nZVI particles have been performed on more than 90 contaminated sites worldwide since 2000.^7^ Compared to more conventional macro/microscale iron, nZVI exhibits substantially increased contaminant removal rates and can be injected into contaminated zones using conventional techniques such as direct push. Laboratory tests and field trials have, however, demonstrated that there are still some obstacles to address in order to reach the full potential of the nZVI technology. These include (i) low electron efficiency of nZVI^8−10^ and (ii) rapid particle agglomeration and sedimentation.^6,11−13^ These limitations reduce the particle reactivity, longevity, and mobility in the subsurface.^14−16^

Several strategies have been investigated to overcome these limitations, such as^17^ (i) doping nZVI with a catalytic noble metal (e.g., Pd, Pt, Ni), (ii) anchoring nZVI onto solid porous materials or modifying its surface via coating with organic polymers and surfactants, and (iii) emulsifying nZVI particles. Recently, the sulfidation of nZVI has attracted great scientific and technical interest due to its beneficial effect on both the particle reactivity with target contaminants and the corrosion resistance.^18^ Even though the above-mentioned strategies largely improved the reactivity and mobility of nZVI particles, they are often associated with some drawbacks. These include the short-lived reactivity of the treated particles (especially if catalytic metals are used), the leaching of the applied catalytic heavy metals, and the increased contaminant sorption to the detriment of chemical reduction in the case of stabilized/supported nZVI.^17,19,20^ At the moment, the sulfidation of nZVI represents a promising approach to enhancing nZVI’s performance, as it does not suffer from these drawbacks.

Nitriding has been known for decades as a useful means of improving the wear, fatigue, and especially corrosion resistance of iron and steel.^21,22^ The exceptional performance of nitrided Fe-based materials spurred research efforts to deploy the nitriding process also for improving nZVI’s properties in groundwater remediation. Nitriding is a thermochemical treatment that consists of the diffusion of nitrogen atoms into interstitial positions of metal lattice, leading to the formation of metal nitrides.^23^ The process typically produces two different layers on the metal surface with different properties.^24^ The outmost layer (compound or “white” layer) in the case of iron contains iron nitrides such as *γ′*-Fe_4_N and *ε*-Fe_2-3_N. The diffusion layer below the compound layer contains a Fe lattice with interstitial N atoms. In contrast, the nitriding of nanocrystalline iron typically results in the formation of iron nitrides in the entire particle volume.^25,26^ The extent of nitriding is governed by the nitriding potential, temperature, and time.^25−28^ Plasma, ammonia gas, or molten salt can be used as sources of nitrogen. Although most synthesis approaches require elevated temperatures, nitriding can be also carried out employing less-energy demanding processes such as cold plasma treatment.^29,30^ The corrosion inhibition by iron nitrides can be attributed to the increase in the corrosion potential resulting from the formation of an anodic passivation layer.^29,31^ Compared to pristine iron, the concurrent higher wear and fatigue resistance is a consequence of the greater hardness of iron nitrides.^32^

Iron nitrides have also been studied as promising (electro)catalysts. In the 1950s, Anderson and co-workers developed iron nitride hydrogenation catalysts for the Fischer–Tropsch synthesis.^33^ Iron nitrides have been found to catalyze ammonia and hydrazine decomposition,^34,35^ amine synthesis,^36^ and oxidative reactions with persulfate.^37^ Various iron nitrides materials were also recognized as promising electrochemical catalysts for water splitting,^38^ oxygen reduction,^39,40^ and CO_2_ reduction.^41^ Moreover, one recent study incorporating Fe–N_*x*_(C) species on the surface of microscale ZVI, which contained mainly pyridinic, pyrrolic, and graphitic N-moieties, led to an increase in the TCE dechlorination rate.^42^

We hypothesized that the high corrosion resistance of iron nitrides combined with their catalytic properties could significantly improve the reactivity and selectivity of nZVI technologies. Unlike catalytic metals used as nZVI amendments, iron and nitrogen are both cheap, nontoxic, and environmentally abundant elements.

To examine this hypothesis, we investigated the performance of nitrided nZVI particles (hereafter referred to as Fe_*x*_N nanoparticles) in the dechlorination of trichloroethylene (TCE) as a model CS and compared it to that of commercially available nZVI. We synthesized Fe_*x*_N nanoparticles by treating commercially available nZVI with gaseous ammonia-nitrogen mixtures at elevated temperatures, investigated the effect of the nitriding extent on the reactivity and longevity of Fe_*x*_N nanoparticles toward TCE dechlorination, and described the dechlorination mechanism by combining theoretical (DFT-based molecular modeling) and experimental approaches.

## 2 Materials and Methods

The preparation of samples for particle characterization, reactivity experiments, and aging was carried out in an Ar-filled glovebox (O_2_ < 30 ppm) unless stated otherwise. Commercially available nZVI particles (type NANOFER 25P^43^) were supplied by NANO IRON (Czech Republic). Two slightly different batches of pristine nZVI were used throughout this study: one for the nitriding procedure (containing 87.2% of α-Fe) and the other one as reference material in the aging and reactivity experiments (containing 93.8% of α-Fe), see Figure S1 and Table S1 in the Supporting Information. All other chemicals were reagent grade and were used as-received. Details on the chemicals used in this study are provided in the Supporting Information (Text S1). Synthetic, moderately hard water (hereafter MHW)^44^ was used for the aging and reactivity experiments after being sparged with N_2_ for 45 min to remove oxygen (dissolved O_2_ concentration < 0.5 mg L^–1^). The composition of deoxygenated MHW with an ionic strength of 4.8 mmol L^–1^ and a pH of 8.2 is given in Table S2, Supporting Information. Ultrapure water used to prepare MHW was obtained from a water purification system (Milli-Q gradient A 10, Millipore, Merck, Germany).

### 2.1 Synthesis of Iron Nitride Nanoparticles

Two types of Fe_*x*_N nanoparticles, encompassing a low and high degree of nitriding (further referred to as *γ′-*Fe_*x*_N and *ε-*Fe_*x*_N, based on the predominant phase), were synthesized according to Arabczyk et al.,^26^ with some minor modifications. Briefly, anhydrous NH_3_/N_2_ gas mixtures (Messer Technogas, Czech Republic) were passed over 50 g of nZVI particles at a pressure of 0.5 bar and temperatures 500 and 300 °C in a fluid laboratory furnace for 3 and 5.5 h, respectively. Further details on nitriding experimental conditions are provided in Table S3, Supporting Information. The flow of NH_3_/N_2_ gas mixtures was maintained to prevent nitride decomposition until the furnace temperature dropped below 250 °C. Subsequently, the furnace was kept in inert conditions under nitrogen until it reached ambient temperature. The particles were transferred into an airtight container and stored under an inert atmosphere inside an Ar-filled glovebox before use.

### 2.2 Particle Characterization

The phase composition and morphology of the freshly synthesized and aged Fe_*x*_N nanoparticles were characterized by X-ray diffraction (XRD), ^57^Fe Mössbauer spectrometry, scanning electron microscopy (SEM), and transmission electron microscopy (TEM), including high-resolution energy dispersive spectrometry (EDS) used for elemental mapping. The hydrophobicity of nanoparticles was determined by water contact angle measurement. Chemical states of Fe and N on the nanoparticle surface were investigated using X-ray photoelectron spectroscopy (XPS). The total Fe and N particle contents were determined by electrothermal atomic absorption spectrometry (AAS) and elemental analysis, respectively. The Fe^0^ content was determined by measuring the volume of hydrogen evolved after the particle acidification. The Brunauer–Emmett–Teller specific surface area (BET SSA), the size distribution of particle agglomerates, and the leaching of inorganic N-containing species after acidification and particle aging were also determined. More details regarding the characterization procedures and the used instruments are described in the Supporting Information (Text S2).

### 2.3 Particle Aging Experiments

The longevity of *γ′-*Fe_*x*_N and *ε-*Fe_*x*_N and their corrosion products in water were determined in a 1 g L^–1^ particle suspension over 104 days. First, particle stock suspensions (20% w/w) were prepared by adding 16 mL of deoxygenated ultrapure water to 4 g of particles and by dispersing them at 11 000 rpm for 2 min using a T25 ULTRA-TURRAX disperser (IKA, Germany). Subsequently, 248 μL of particle stock suspension was spiked into 120 mL serum bottles containing 60 mL of deoxygenated MHW. The bottles were capped with FEB-faced chlorobutyl-isoprene septa, taken out of the glovebox, and placed on a horizontal shaker (125 rpm) at 22 ± 1 °C for the whole duration of aging. Aging experiments were done in four replicates, two of which were used for particle characterization and two for consecutive reactivity experiments. At regular intervals, overpressure was manually released and recorded using a frictionless glass syringe (Poulten & Graf Ltd., Germany). Good reproducibility of a pressure buildup among the replicates was observed, confirming no significant gas losses. After approximately three months of aging, the particle morphology and composition were investigated, including the leaching of inorganic nitrogen compounds into the MHW. Control experiments with pristine nZVI were performed in parallel.

### 2.4 TCE Dechlorination Experiments

The ability of fresh and aged Fe_*x*_N particles to dechlorinate TCE, as well as the concentrations of ethane, ethene, acetylene, chloride, and hydrogen, were determined using a previously established method,^45^ with some minor modifications. A nontarget headspace analysis was performed using a gas chromatograph coupled to a high-resolution quadrupole time-of-flight mass spectrometer at the end of the dechlorination experiment with fresh *γ′-*Fe_*x*_N nanoparticles to investigate the full range of TCE dechlorination products. A detailed description of the method is provided in the Supporting Information (Text S3).

### 2.5 Molecular Modeling

Density functional theory (DFT) calculations in periodic boundary conditions were performed on a TCE molecule in a gas phase and on the *γ′-*Fe_4_N(001) and the α-Fe(110) surfaces to investigate the role of the Fe_4_N and Fe surfaces in facilitating the dechlorination reaction. To study the effect of N and S atoms on the hydrophobicity of Fe-bearing minerals, adsorption energies of water on the α-Fe(110), *γ′-*Fe_4_N(001), and FeS(001) surfaces were calculated. The methods are described in detail in the Supporting Information (Text S4).

## 3 Results and Discussion

### 3.1 Nitriding Results in a Uniform Nitrogen Diffusion into nZVI Particles, with Increased Surface Availability of Reduced Iron

**Figure 1:**
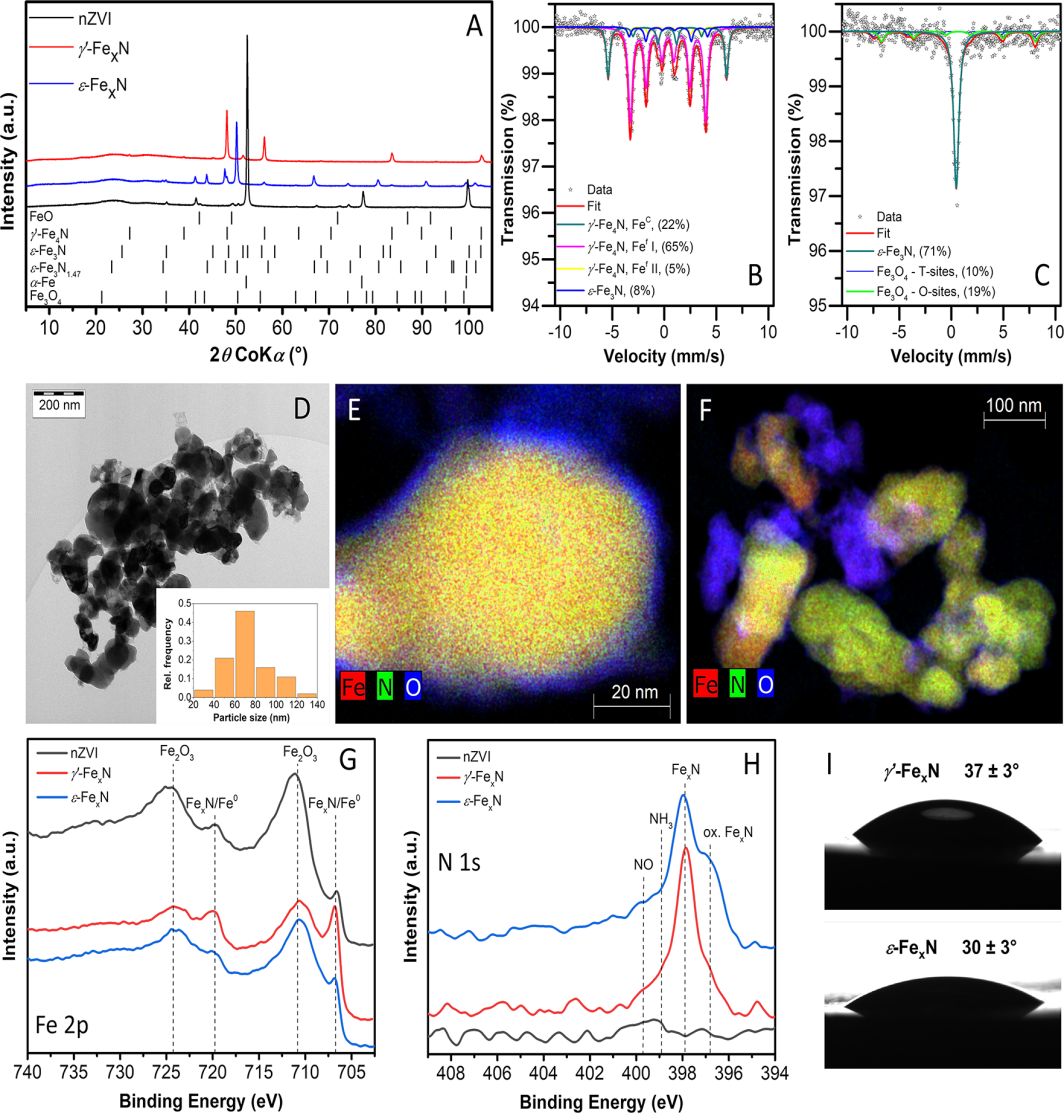
Material characterization of fresh Fe_*x*_N and pristine nZVI particles: (A) XRD patterns, (B) ^57^Fe Mössbauer spectrum of *γ′-*Fe_*x*_N recorded at 150 K, (C) ^57^Fe Mössbauer spectrum of *ε-*Fe_*x*_N recorded at 150 K, (D) TEM image of a *γ′-*Fe_*x*_N agglomerate with inserted particle-size distribution, (E) STEM EDS overlay of Fe–N–O mapping of a *γ′-*Fe_*x*_N particle, (F) STEM EDS overlay of Fe–N–O mapping of an *ε-*Fe_*x*_N particle agglomerate, (G) Fe 2p XPS narrow region spectra, (H) N 1s XPS narrow region spectra, and (I) water contact angles on dry pellets of *γ′-*Fe_*x*_N and *ε-*Fe_*x*_N particles in the air.

Pristine nZVI particles, before nitriding, consisted of ∼90% α-Fe, with characteristic 2θ peaks at 52.5° and 99.5° on the XRD pattern (Figure 1A and Table S4). Magnetite (Fe^2+^Fe^3+^_2_O_4_) and wüstite (FeO) were detected as minor nonreduced phases.^43^ The formation of Fe_*x*_N phases during nitriding was controlled by nitriding temperature, time, and nitriding potential.^25,26,28^ The XRD pattern of freshly prepared iron nitride nanoparticles revealed *γ′-*Fe_4_N as the dominant crystalline phase (>90%) in the particles nitrided at 500 °C for 3 h. Particles nitrided at 300 °C for 5.5 h contained mostly nonstoichiometric *ε-*Fe_2–3_N (84.4%) (Figure 1A and Table S4). These two particle types are hereafter referred to as *γ′-*Fe_*x*_N and *ε-*Fe_*x*_N, respectively. The dominant Fe_*x*_N phases obtained by nitriding at different temperatures and times are in agreement with a previous study.^26^ Low amounts of other Fe_*x*_N phases were detected in both particle types: *ε-*Fe_2–3_N (9.3%) in *γ′-*Fe_*x*_N and *γ′-*Fe_4_N (4.1%) in *ε-*Fe_*x*_N. Iron oxide impurities, identified in pristine nZVI particles, were completely reduced during nitriding at 500 °C, while nitriding at 300 °C led only to the reduction of wüstite, resulting in the presence of magnetite (11.7%) in the *ε-*Fe_*x*_N particles.

The phase composition of two fresh Fe_*x*_N nanoparticle types was further confirmed using low-temperature ^57^Fe Mössbauer spectroscopy. The *γ′-*Fe_*x*_N spectrum contained four sextet spectral components (Figure 1B, Table S5). Three sextet components represent the nonequivalent Fe cation sites in cubic *γ′-*Fe_4_N.^46,47^ The fourth sextet with a hyperfine magnetic field of 23.4 T indicates the presence of *ε-*Fe_3_N,^23^ which is in full accordance with the result of XRD analysis (Figure 1A). In contrast, the ^57^Fe Mössbauer spectrum of the *ε-*Fe_*x*_N particles shows two distinct sextet components and one doublet component (Figure 1C). Based on the values of the Mössbauer hyperfine parameters (Table S5), the sextet with the higher magnetic hyperfine field can be ascribed to the Fe^3+^ ions located in the tetrahedral sites of magnetite, while the sextet with the lower values of the hyperfine magnetic field belongs to the Fe^2+^ and Fe^3+^ ions occupying the octahedral sites in a magnetite spinel crystal structure.^48^ The doublet component can be assigned to the nonstoichiometric *ε-*Fe_2–3_N in superparamagnetic ordering^49−51^ (i.e., with low-temperature superparamagnetic transition observed on magnetization data; unpublished data of authors). This feature, different from the *γ′*-Fe_*x*_N spectrum, could be explained by a domain structure of the *ε*-Fe_*x*_N particles with a low degree of stoichiometry due to nitriding at a lower temperature.

All particle types, including precursor nZVI, formed ca. 1–3 μm large particle agglomerates (Figures 1D, S2A–C, S3, and S4 and Table S6). Individual particles were roughly spherical with an average particle size of ∼75 nm (Table S7). The size of the Fe_*x*_N particles was not significantly affected by nitriding. High-resolution STEM-EDS elemental mapping revealed a uniform nitrogen distribution within Fe_*x*_N particles (Figures 1E and 1F). This is in agreement with XRD and ^57^Fe Mössbauer spectroscopy characterizations, showing that nitrogen diffused throughout the entire volume of the nZVI particles, thus forming distinct bulk Fe_*x*_N phases. In the elemental mapping of the *ε-*Fe_*x*_N sample (Figure 1F), two different types of particles can be observed–one with and one without nitrogen. While the first particle type corresponds to *ε*-Fe_*x*_N particles, the other type is likely magnetite, an artifact from the nZVI synthesis identified by both XRD and Mössbauer spectroscopy.^43^ Elemental mappings for individual elements are provided in the Supporting Information (Figures S5 and S6). STEM-EDS also revealed a thin oxygen-rich layer on the particle surface (Figure 1E). Fe_*x*_N particles, especially *γ′-*Fe_*x*_N, exhibited a thinner and more compact surface (oxyhydr)oxide shell, compared to pristine nZVI (*p* < 0.005, Figure S2D–F and Table S7). This is in line with previous studies showing that upon nitriding, a thin stable Fe^3+^ (oxyhydr)oxide layer with very low oxygen and ion diffusion coefficients is formed on the nitrided surface, thus inhibiting further corrosion.^29−31^

To shed more light on the surface properties of Fe_*x*_N particles, XPS survey scans and high-resolution spectra were collected. Survey scans indicate that O, Fe, and N were the most abundant elements on the surface of the particles (Figure S7 and Table S8). The Fe 2p high-resolution spectra of all particle types were deconvoluted into two components with characteristic Fe 2p_3/2_ binding energies of 706.8 and 710.8 eV, corresponding to Fe_*x*_N/Fe^0^ and Fe^3+^ occurring in iron oxides (Figures 1G and S8).^52,53^ It is worth noting that the spectral shape and binding energy of elemental Fe are almost identical to those of Fe_*x*_N, and therefore, they cannot be well distinguished from each other.^52−55^ This can be explained by only a small positive charge of Fe in *γ′-*Fe_4_N and *ε-*Fe_2-3_N (0.2–0.5 |e|), which results in a character similar to zerovalent iron.^56^ Most importantly, the nitriding of nZVI had a considerable effect on the surface availability of iron in a reduced form, as the relative intensity of the Fe_*x*_N/Fe^0^ peaks increased in order nZVI < *ε-*Fe_*x*_N < *γ′-*Fe_*x*_N (Table S9). This is in line with the higher corrosion resistance of the Fe_*x*_N phases, compared with Fe^0^ and a thinner (oxyhydr)oxide layer on the Fe_*x*_N particle surface.^29−31^ The N 1s spectra of Fe_*x*_N particles contained four components at 396.8, 397.9, 398.9, and 399.7 eV (Figures 1H and S9). The first two correspond to oxidized Fe_*x*_N and pristine Fe_*x*_N, respectively.^53,57,58^ The other two spectral lines can be attributed to adsorbed ammonia and NO species.^30,31,53,57^ Both species are likely to be present on the particle surface in small amounts given that ammonia was used as the nitrogen source in the nitriding process and the NO species are typically detected on the surface of nitrided metals.^30,31,53^ The line attributed to oxidized Fe_*x*_N was more pronounced in *ε-*Fe_*x*_N than in *γ′-*Fe_*x*_N, implying higher surface oxidation of the *ε-*Fe_*x*_N particles (Table S9), which likely stemmed from the incomplete reduction of magnetite originally present in the precursor nZVI. This is in agreement with a lower abundance of reduced iron on the *ε-*Fe_*x*_N particle surface, compared with *γ′-*Fe_*x*_N.

Water contact angle measurements indicate that both Fe_*x*_N particle types (contact angles 30–37°, Figure 1I) were less hydrophilic than pristine nZVI (contact angle ∼18°).^59^ The measured water contact angle of *γ′-*Fe_*x*_N particles was slightly higher than that of *ε-*Fe_*x*_N particles, likely due to the higher surface oxidation of the latter. A similar contact angle was previously measured for sulfidated nZVI (S-nZVI) prepared using the postsulfidation approach (contact angle ∼36°), while cosulfidated S-nZVI exhibited substantially higher hydrophobicity (contact angle ∼103°).^59^ This implies that sulfidation of nZVI has a more profound effect on particles’ hydrophobicity than nitriding, as predicted from theoretical calculations discussed below.

The BET SSA, another crucial surface parameter of reactive nanoparticles, was not significantly affected by nitriding in the case of the *ε-*Fe_*x*_N particles, whereas the *γ′-*Fe_*x*_N particles exhibited about a 17% decrease in SSA, compared with pristine nZVI (Table S10). This is probably a result of the recrystallization of the *γ′-*Fe_*x*_N particle surface due to high temperature during the synthesis (500 °C) and/or the different surface properties of the predominant Fe_*x*_N phases in the two Fe_*x*_N particle types.^31^

Depending on the nitriding protocol, the average N content ranged from 5.3% in *γ′-*Fe_*x*_N to 7.6% in *ε*-Fe_*x*_N (Table S11), which coincided with the higher relative amount of N-rich phases (*ε*-Fe_2_N and *ε*-Fe_3_N) in the latter particle type, according to the XRD and Mössbauer data (Figure 1A–C). As expected, the nitriding of the nZVI particles resulted in a lower Fe content in the nanoparticles (i.e., from 99.3% in pristine nZVI to 96.2% and 91.2% in *γ′-*Fe_*x*_N and *ε-*Fe_*x*_N particles, respectively). The increase in the nitrogen content was accompanied by a drop in the particle reducing capacity (Table S11). This might be a consequence of the redox processes between iron and atomic nitrogen, in which nitrogen is reduced to nitride.

### 3.2 Degree of Nitriding Controls the Longevity of Fe_*x*_N Nanoparticles in Aqueous Environments

**Figure 2:**
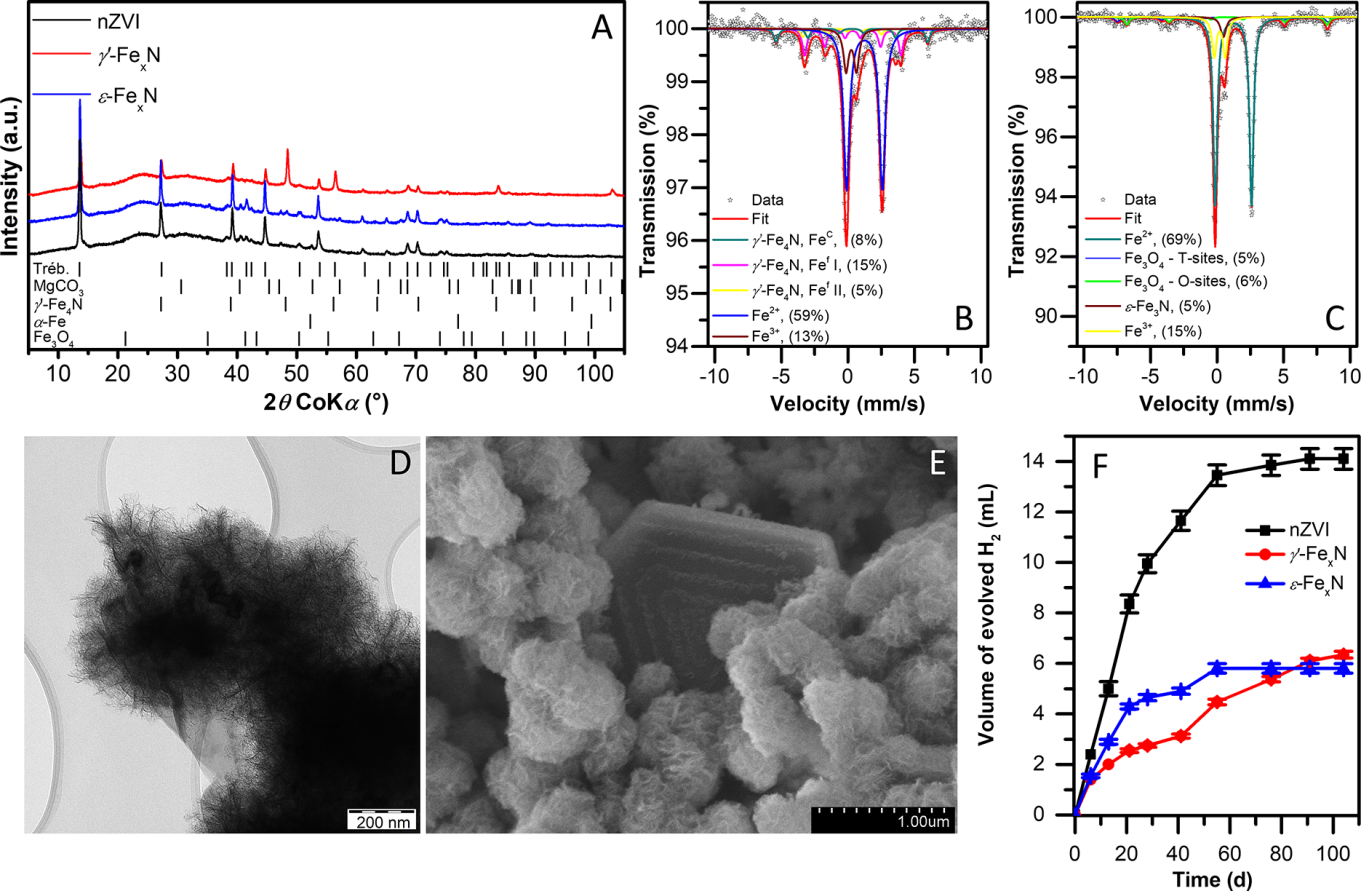
Material characterization of Fe_*x*_N and pristine nZVI particles aged three months in MHW: (A) XRD patterns, (B) ^57^Fe Mössbauer spectrum of *γ′-*Fe_*x*_N recorded at 150 K, (C) ^57^Fe Mössbauer spectrum of *ε-*Fe_*x*_N recorded at 150 K, (D) TEM image of *γ′-*Fe_*x*_N, (E) SEM image of *γ′-*Fe_*x*_N, and (F) hydrogen evolution during aging.

The characterization of nanoparticles recovered from three-month-aged suspensions revealed that the extent of nitriding (and/or atomic structure of particular Fe_*x*_N phases) affected the particle longevity. The observed corrosion in MHW was slower for the *γ′-*Fe_*x*_N particles as compared with *ε-*Fe_*x*_N and pristine nZVI (Figure 2A–C). Based on the XRD patterns, the *γ′-*Fe_*x*_N particles still contained about 40% of a crystalline *γ′*-Fe_4_N fraction after aging, whereas the *ε-*Fe_*x*_N and pristine nZVI contained only 2.5% and 1.1% of reduced iron phases (i.e., Fe_*x*_N and/or *α*-Fe), respectively (Table S4). This was corroborated by the ^57^Fe Mössbauer spectroscopy. *γ′-*Fe_*x*_N contained three clear sextets, assigned to three nonequivalent Fe cation sites in cubic *γ′*-Fe_4_N,^46^ representing 28% of iron-containing phases. A fraction of iron nitrides (*ε-*Fe_3_N) was also preserved in aged *ε-*Fe_*x*_N particles, but their abundance (5%) was much smaller than in aged *γ′-*Fe_*x*_N (Table S5).^49−51^ This contradicts previous findings that corrosion resistance increases with increased nitrogen content.^29,31^ It is important to bear in mind that this trend was previously observed for macroscopic nitrided metal surfaces and may not be directly transferable to nanoparticles nitrided in their entire volume.

The most abundant corrosion product detected for all particle types was the carbonate green rust mineral trébeurdenite [Fe^2+^_2_Fe^3+^_4_O_2_(OH)_10_][CO_3_]·3H_2_O, a common iron corrosion product in anoxic carbonate-containing waters.^60^ Based on the XRD patterns, trébeurdenite represented 90.7%, 78.2%, and 59.9% crystalline phases in aged nZVI, *ε-*Fe*_x_*N, and *γ′-*Fe_*x*_N samples, respectively (Table S4). This is in line with the ^57^Fe Mössbauer spectra of both aged Fe_*x*_N particle types containing a dominant wide doublet corresponding to Fe^2+^ ions in the crystal structure of green rust minerals^61^ accompanied by a narrow doublet, which can be attributed to the Fe^3+^ ions occupying the green rust octahedral sites.^61^ Overall, these multiplets represent 84% and 72% of iron-containing phases in *ε-*Fe*_x_*N and *γ′-*Fe_*x*_N samples, respectively (Table S5).

The aging of all particle types resulted in similar morphological changes. Flakes of iron (oxyhydr)oxides coating primary nanoparticles were apparent in all TEM and SEM images (Figures 2D, 2E, and S10). Distinct hexagonal platelets of carbonate green rust^62^ were clearly visible in SEM images (Figures 2E and S10), as well. Both microscopic techniques evidenced an increase in the particle agglomerate size (Figures S2, S3, and S10), which was corroborated with laser diffraction analysis (Figure S4 and Table S6). The median of the particle size distribution (*d*_50_) increased in the following order: *γ′-*Fe_*x*_N < *ε-*Fe_*x*_N < nZVI, which is consistent with the agglomerate size distribution of fresh particles. Apparently, nitriding has a slightly inhibiting effect on particle agglomeration. Slower Fe_*x*_N corrosion compared to pristine nZVI may have further reduced the growth of particle agglomerates during aging.

The corrosion of all particle types was accompanied by the hydrogen evolution reaction (HER). The rate of the HER of both Fe_*x*_N particle types was considerably lower during aging than that of pristine nZVI (Figure 2F), corroborating a higher corrosion resistance of Fe_*x*_N. Interestingly, *γ′-*Fe_*x*_N particles evolved H_2_ at a slower rate than *ε*-Fe_*x*_N, which contradicts previous findings that corrosion resistance increases with increased nitrogen content.^29,31^ At the end of the aging experiments, an ongoing H_2_ evolution was observed for the *γ′*-Fe_*x*_N particles, while the volume of evolved H_2_ did not further increase for the *ε-*Fe_*x*_N and nZVI particles, indicating the depletion of the particle reducing capacity and/or surface passivation (Figure S11). As documented by the particle reducing capacity measurements (Table S11), fresh *ε-*Fe_*x*_N retained only about 25% of the reducing capacity compared to precursor nZVI. Therefore, even though *ε-*Fe_*x*_N corroded more slowly than nZVI, its reducing capacity was quickly depleted. The evolved H_2_ volume in the *ε*-Fe_*x*_N samples indeed reached levels similar to the amount of H_2_ evolved during the HCl digestion of fresh *ε*-Fe_*x*_N particles, implying complete particle oxidation. This is in line with the particle characterization and complete leaching of nitrogen, as described below. Apparently, there is a trade-off in nitriding between the increased corrosion resistance and the lowered particle reducing capacity. The composition of *γ′-*Fe_*x*_N may be closer to the optimal nitriding extent (or structural form) as its longevity was substantially higher. The detected H_2_ volume in aged nZVI samples corresponded to depletion of only 2/3 of the nZVI reducing capacity. The formation of a passivating layer of iron corrosion products on the particle surface was likely responsible for the observed nZVI passivation, rather than the reducing capacity depletion.^63^

To further investigate the fate of nitrogen in the course of the particle aging, the concentrations of dissolved NH_3_, NO_2_^–^, and NO_3_^–^ were determined in aged suspensions (Table S12). Interstitial nitrogen atoms were found to leach into the solution as ammonia. Ammonia levels in aged *γ′*-Fe_*x*_N and *ε-*Fe_*x*_N suspensions reached 35.8 mg L^–1^ and 79.5 mg L^–1^, accounting for about 68% and 100% of nitrogen initially present in the particles, respectively. These findings are in agreement with the abundance of Fe_*x*_N phases in aged nanoparticles, as documented by XRD and Mössbauer spectroscopy (Figure 2A–C). The gradual release of ammonia at low levels (<0.5 g L^–1^) in groundwater could increase the efficiency of combined biotic-abiotic CE treatments as the addition of the exogenous nitrogen source stimulates reductive dechlorination by *Dehalococcoides*.^64^

### 3.3 Even after Three Months of Aging, Fe_*x*_N Nanoparticles Dechlorinate TCE 20 Times Faster than nZVI

**Figure 3:**
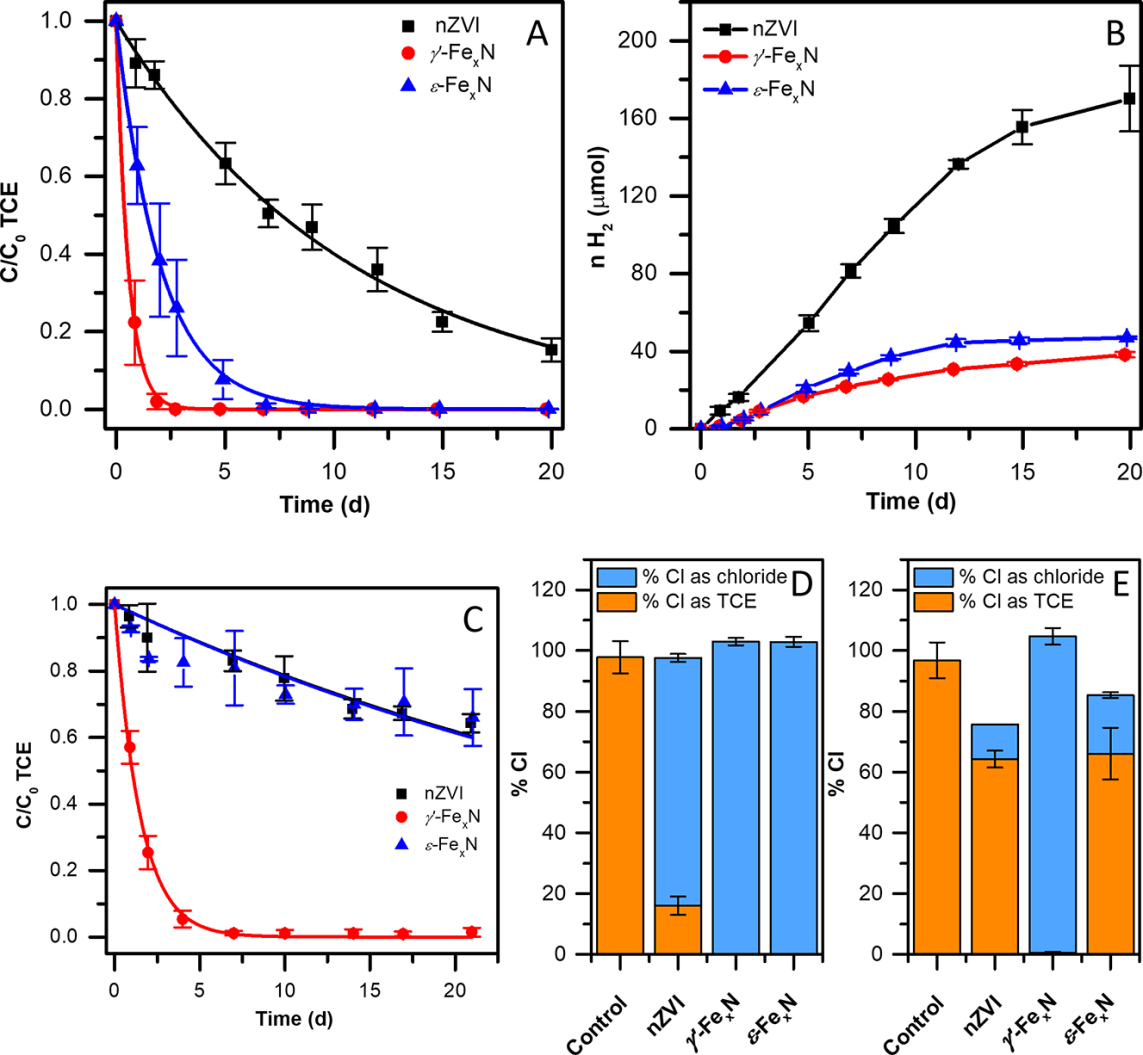
(A) TCE removal by fresh Fe_*x*_N and nZVI particles; (B) hydrogen production by fresh particles during the TCE degradation experiment; (C) TCE removal by Fe_*x*_N and nZVI particles aged for three months; (D) and (E) chlorine balance for experiments with fresh and aged particles, respectively. The reactions were carried out at an initial TCE concentration of 20 mg L^–1^ and particle concentration of 1 g L^–1^. Whiskers indicate standard deviation (SD).

Both types of fresh Fe_*x*_N nanoparticles showed remarkably high rates of TCE reduction: the observed pseudo-first-order reaction rate constants (*k*_obs_) of *γ′-*Fe_*x*_N and *ε*-Fe_*x*_N were roughly 20- and 5-fold higher, respectively, than those of conventional nZVI particles (Figure 3A and Table S13). As the SSAs of all fresh particle types were comparable (18.9–23.2 m^2^ g^–1^), a similar trend was apparent also for the surface-area normalized rate constants (*k*_SA_). In contrast, the rate of HER was substantially lower for both Fe_*x*_N particle types (Figure 3B). The initial zero-order HER rate constants of *γ′-*Fe_*x*_N and *ε*-Fe_*x*_N, calculated from the linear portion of the curves (*t* < 9 days), were 3-fold lower than those of unmodified nZVI (Table S13). The observed TCE removal and HER rates show that the nitriding of nZVI particles dramatically increases their reactivity and electron selectivity. Thus, the effect of nitriding is comparable to that of sulfidation (Figure S12). Particle longevity estimated from the particle initial reducing capacity and the amount of hydrogen gas evolved during the 3 weeks of reaction was three times higher for *γ′*-Fe_*x*_N than for pristine nZVI (Table S13). Interestingly, no significant decrease in the HER rate was observed when microscale ZVI was ball-milled with melamine.^42^ Apparently, crystalline Fe_*x*_Ns are needed to inhibit the HER. The addition of catalytic metals, as opposed to nitriding and sulfidation, leads to the accelerated corrosion of nZVI, which results in poor longevity and overall performance under particle excess conditions.^19,20,65,66^

Particles aged for three months displayed a different reactivity pattern (Figure 3C). The TCE dechlorination rate of the *γ′-*Fe_*x*_N particles was almost unaffected by aging (Table S13), reaching a complete TCE dechlorination in about 5 days. Only 43% of the *γ′-*Fe_*x*_N reducing capacity was depleted over 104 days (Figure S11), which is remarkably similar to the reported ∼50% drop in the reducing capacity of S-nZVI after 120 days of aging.^67^ It should be noted, however, that in the cited study, S-nZVI was aged inside a glovebox under static conditions, while our aging experiments were performed on a shaker. It is reasonable to assume that the longevity of S-nZVI under dynamic conditions would be lower. In contrast to the high longevity of *γ′-*Fe_*x*_N, aged *ε*-Fe_*x*_N degraded TCE slower by a factor of 20, compared to its fresh counterpart, at approximately the same rate as aged nZVI particles. A decrease in reactivity during aging can be explained by particle corrosion, which depletes the particle reducing capacity and forms a surface passivation layer.^9,10,63,68^ The notable decrease in the reactivity of *ε*-Fe_*x*_N likely stems from the depletion of its reducing capacity, as discussed above (Figure S11). Even though both types of Fe_*x*_N particles exhibited a limited HER (Figures 2F and 3B), the *ε*-Fe_*x*_N particles had a lower initial reducing capacity (Table S11). Therefore, we assume that there is an optimal extent of particle nitriding (and/or structural arrangement of Fe_*x*_N on the particle surface) at which the Fe_*x*_N reactivity and longevity can be maximized; the composition of *γ′*-Fe_*x*_N particles may be close to such an optimum. The low reactivity of aged nZVI was attributed to surface passivation, as described above.

Chlorine balance was determined at the end of the reactivity experiments to control whether a complete TCE dechlorination was achieved. For fresh particles, the amount of total chlorine corresponded to the initial amount injected as TCE for all tested particle types (Figure 3D). This implies that no significant amounts of chlorinated byproducts were formed during the TCE dechlorination by the Fe_*x*_N particles (see below). It also indicates that TCE losses due to leakage and sorption on the particles’ surface were negligible. In experiments with aged particles, a complete TCE dechlorination to chloride was observed only for the *γ′*-Fe_*x*_N particles, whereas *ε*-Fe_*x*_N and nZVI reached a chlorine balance of only 85.3% and 75.7%, respectively (Figure 3E). As these two particle types underwent passivation during aging, chlorine remained predominantly bound as TCE, leading to an incomplete chlorine balance due to increased TCE sorption to iron and its corrosion products.^63^

### 3.4 TCE Was Reduced to Aliphatic Hydrocarbons

Ethene and ethane were the major C_2_ dechlorination products in the experiments with fresh Fe_*x*_N particles (Figure S13). Trace amounts of *cis*-1,2-dichloroethene and 1,1-dichloroethene were also detected for all particle types (<1% of the original amount of TCE),^69^ while neither vinyl chloride nor *trans*-1,2-dichloroethene was observed. This product pattern is consistent with the reductive *β*-elimination pathway.^69,70^ While the C_2_-carbon recovery at the end of the reactivity experiments with fresh particles was 69.1% for pristine nZVI, only 16.2% and 42.9% were achieved for the *γ′*-Fe_*x*_N and *ε*-Fe_*x*_N particles, respectively. The decrease in the C_2_-carbon recovery for the Fe_*x*_N particles is due to a more noticeable production of the C–C coupling products, which are probably formed through the Fischer–Tropsch-type reactions catalyzed by Fe_*x*_N species.^33,71^ In a typical Fischer–Tropsch process, carbon in CO is hydrogenated into CH_2_ species that polymerize into a hydrocarbon chain.^72^ Nontarget headspace analysis conducted at the end of the reactivity experiment with fresh *γ′-*Fe_*x*_N nanoparticles tentatively identified several longer-chain hydrocarbons (Table S14). Similarly, a more pronounced formation of longer-chain hydrocarbons was observed when TCE was dechlorinated by microscale ZVI amended with melamine.^42^ All degradation products identified using the nontargeted approach were only aliphatic hydrocarbons, while neither aromatic moieties nor organic nitrogen or chlorine was observed. Although precise identification and quantification of all products of TCE dechlorination by the Fe_*x*_N particles was outside the scope of this study, it can be reasonably anticipated that the reaction products are of much lower environmental concern than TCE.

The aging of nanoparticles did not affect substantially the product pattern of TCE dechlorination by the *γ′*-Fe_*x*_N particles. However, aged *ε*-Fe_*x*_N and pristine nZVI (Figure S13) evolved only small quantities of products, notably acetylene. This shift in the product composition can be attributed to particle passivation, which hindered the generation of reactive hydrogen on the particle surface and, consequently, led to a decreased reactivity and preference for less-reduced products.^73^

### 3.5 Fe_*x*_N Surface Facilitates TCE Dechlorination, and Its Slower Corrosion Is Decisive for Improved Performance

**Figure 4:**
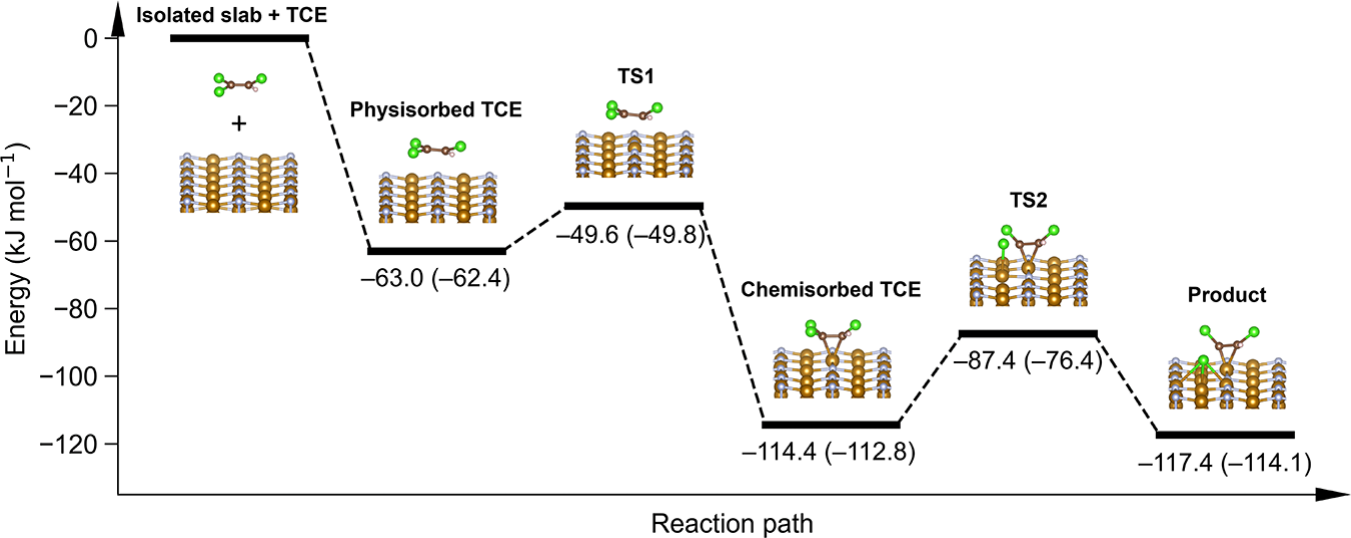
Energy profiles of TCE adsorption and the first C–Cl cleavage on the *γ′*-Fe_4_N(001) surface. TS denotes transition state. The reported energy values were calculated with an implicit solvent model and in the gas phase (values in parentheses).

DFT calculations were employed to elucidate the mechanism of TCE dechlorination by the Fe_*x*_N particles. These calculations were performed in the gas phase and in a solvent (water), which was represented by an implicit solvation model developed for solid–liquid interfaces^74,75^ (see Text S4 for details). Given that the *γ′*-Fe_4_N phase was dominant in the *γ′*-Fe_*x*_N particles, we constructed a periodic slab model based on known crystallographic data of the *γ′*-Fe_4_N structure^76^ (details given in the Text S4). In contrast, the exact stoichiometry of the ε-Fe_2–3_N phase, this being the dominant phase in the *ε-*Fe_*x*_N particles, was not known, and therefore, it was not possible to create a realistic model. The DFT calculations showed that TCE physisorbed on the *γ′*-Fe_4_N(001) surface with its main molecular plane arranged parallel to the surface. The calculated TCE adsorption energy, *E*_ads_, was −63.0 kJ mol^–1^ with the inclusion of solvent, which was only slightly different from the gas phase adsorption energy of −62.4 kJ mol^–1^ (Figure 4). The small difference illustrates that the solvent has only a negligible effect on TCE adsorption. As the same trend was typically observed for consecutive reaction steps, we discuss below only results of the calculations with the implicit solvent. The only reaction step with a significant solvent effect was the first TCE dechlorination reaction as discussed below. In the adsorption complex, the C=C bond was localized above a Fe atom of the top Fe_II_N layer at a perpendicular distance of ∼3.3 Å (Figure S14). The atop site has been previously found to be the most energetically favorable for TCE adsorption on the Fe(110) surface.^77^

In the next step, TCE transitioned into a more stable chemisorbed configuration (*E*_ads_ = −114.4 kJ mol^–1^) after surpassing a small energy barrier (*E*^‡^_TS1_, Figure 4) of 13.4 kJ mol^–1^. The C=C bond approached the atop Fe atom to ∼2 Å (Figure S14). The stabilization was reached mainly due to the strong interaction of the π-bond with a surface Fe atom.^77^ As a result, the geometry of the chemisorbed TCE molecule was deformed. The Cl–C–C–Cl and Cl–C–C–H dihedral angles decreased from 180.0° to 136.8° and 139.2°, respectively (Table S15). The C=C and C–Cl bonds were elongated by 0.06 to 0.10 Å (Table S15). Such a distorted geometry was associated with a strong activation toward dechlorination reactions on the Fe(110) surface.^77,78^

Only the dissociation of one chlorine atom from TCE yielding *cis-*1,2-dichloroethene and Cl radicals (homolytic C–Cl dissociation) was further considered since this C–Cl bond has the lowest bond dissociation energy (BDE) in the gas phase (Table S16). Moreover, this reaction was previously identified as the TCE dechlorination rate-liming step on the Fe surface.^78^ The virtual absence of less chlorinated degradation products confirmed that the first C–Cl bond cleavage was the rate-limiting step. While the C–Cl BDE of the isolated TCE molecule was at least 380 kJ mol^–1^ (Table S16), the *γ′*-Fe_4_N(001) surface was found to reduce the first step dechlorination energy barrier (*E*^‡^_TS2_, Figure 4) almost 15-fold, to 27.0 kJ mol^–1^, with the inclusion of solvent. In this reaction step, the solvation led to a stabilization of the transition state (*E*^‡^_TS2_ in the gas phase calculation reached 36.4 kJ mol^–1^). The overall energy profile of the adsorption and dechlorination steps obtained by the climbing image nudged elastic band method is shown in Figure 4. As the rate of the dechlorination reaction is directly proportional to exp(−*E*^‡^_TS2_/*RT*), lowering the activation barrier ∼15-fold increases the reaction rate by many orders of magnitude. After the first chlorine atom is cleaved from the CCl_2_ group, several consecutive steps can follow. In particular, a second C–Cl cleavage is supposed to occur at the CHCl group (*β*-elimination), yielding chloroacetylene.^69,70,78^ Chloroacetylene is very reactive and rapidly undergoes further dechlorination via hydrogenolysis, hydrogenation, and/or the rearrangement of C–C bonds (Figure S15).^69^

Our efforts to calculate the energy profile of TCE dechlorination on the pristine α-Fe surface led to a spontaneous detachment of chlorine atoms and the formation of chemisorbed chloroacetylene during the full geometry relaxation (Figure S16), which did not allow the calculation of energy barriers. Spontaneous TCE dechlorination on various Fe surfaces has been reported previously.^79,80^ Energy barriers of TCE sequential dechlorination reactions on the α-Fe(110) surface were previously calculated only by using lax convergence criteria to obtain a nondissociated adsorption complex and by freezing all but one dissociating Cl atom in the calculations.^77,78^ This may suggest that Fe should exhibit the same or even higher reactivity with TCE than Fe_4_N. In realistic scenarios, however, direct contact between the contaminant molecule and the pristine nZVI surface is practically unattainable because of the fast nZVI corrosion in water, which results in the formation of a surface layer of iron (oxyhydr)oxides.^63,81^ Thus, the driving factor of the higher Fe_*x*_N reactivity with TCE is likely the character and thickness of the particle passivation layer. It is known that upon nitriding, a very thin but stable Fe^3+^ (oxyhydr)oxide layer is formed on the nitrided surface, inhibiting further corrosion.^29−31^ This is in line with a thinner (oxyhydr)oxide shell observed on the Fe_*x*_N particles (Table S7) and the increased availability of iron in a reduced form, i.e., as Fe_*x*_N, on their surface, compared with pristine nZVI (Figures 1G and S8 and Table S9). Additional factors contributing to improved Fe_*x*_N performance over pristine nZVI include faster electron transfer from reduced Fe species to adsorbed contaminants across the thinner (oxyhydr)oxide surface layer^63^ and lower affinity to water molecules as evidenced by water contact angle measurements and DFT calculations (Figure S17 and ref (82)). Similar to S-nZVI, Fe_*x*_N particles with an appropriate extent of nitriding are more resistant to corrosion and reducing capacity depletion than pristine nZVI, and they are expected to provide extended availability of reactive (nonpassivated) surfaces for a longer period. To completely understand how the extent of nitriding affects the Fe_*x*_N reactivity, more realistic surface models such as those involving oxidized Fe_*x*_N/Fe surfaces and surfaces with various Fe/N stoichiometry are needed. The findings presented here may serve as the first step toward a mechanistic understanding of the TCE removal on more complex Fe_*x*_N surfaces.

## 4 Implications for Water Treatment

In this study, we demonstrated that Fe_*x*_N nanoparticles with an appropriate extent of nitriding represent new and potentially important agents for groundwater remediation with the capability of overcoming many limitations of the current nZVI-based technologies. Similar to S-nZVI, Fe_*x*_N nanoparticles dechlorinate TCE much faster and, at the same time, are less prone to corrosion in water, compared to conventional nZVI, which results in better contaminant selectivity and higher particle longevity. These characteristics are crucial for the field-scale application as a higher contaminant removal is anticipated by a unit mass of particles, and consequently, fewer particle injections will be needed on site to reach remediation goals.

Fe_*x*_N nanoparticles degraded TCE to ethane, ethene, and a mixture of longer-chain aliphatic hydrocarbons. The reaction steps involved in TCE reductive dechlorination are analogous to ZVI materials, i.e., *β*-elimination followed by hydrogenolysis and hydrogenation. Although not all TCE dechlorination products could have been identified and quantified in the present study, the absence of halogen, nitrogen, and aromatic moieties in their structure, as evidenced by the nontarget analysis and complete chlorine balance, demonstrates a much lower environmental concern compared to the carcinogenic TCE. Moreover, the produced hydrocarbons and/or their transformation products could be consumed in the subsurface by dehalorespiring bacteria as a carbon source stimulating their growth.^83^ Given the similarity between hydrocarbon products of TCE dechlorination using Fe_*x*_N and products of the Fischer–Tropsch process, the observed products could eventually be recovered as precursors to value-added chemicals such as fuels.^72^

To further improve the performance and applicability of Fe_*x*_N nanoparticles in remediation, future studies should focus on the careful optimization of the nitrogen content and distribution within particles, as well as on the use of cost-effective and environmentally friendly approaches to nZVI nitriding, such as cold plasma treatments. Future research should also critically compare the stability of nitrided and sulfidated nZVI under various particle injection conditions and groundwater composition.

## Supporting Information Available

Additional information on chemicals, materials, and methods; characterization data of fresh and aged particles including XRD, Mössbauer, elemental composition, BET surface area, SEM and TEM images, particle size and shell thickness, STEM-EDS mappings, and XPS; depletion of reducing capacity during aging; concentrations of leached nitrogen species; dechlorination rates, hydrogen evolution rates, and rates of C_2_-hydrocarbons formation; comparison with fresh S-nZVI; tentatively identified dechlorination products; structural models of physisorbed and chemisorbed TCE; TCE geometries and bond dissociation energies; H_2_O adsorption energies and configurations; and proposed reaction mechanism (PDF)

## Notes

The data from DFT calculations underlying this study are openly available in Zenodo at DOI: 10.5281/zenodo.6338412.

## References

[ref1] Panagos P.; Van Liedekerke M.; Yigini Y.; Montanarella L. Contaminated Sites in Europe: Review of the Current Situation Based on Data Collected through a European Network. J. Environ. Public Health 2013, 2013, 1–11. 10.1155/2013/158764. PMC3697397 23843802

[ref2] McCarty P. L. Groundwater Contamination by Chlorinated Solvents: History, Remediation Technologies and Strategies. In In Situ Remediation of Chlorinated Solvent Plumes; Stroo H., Ward C., Eds.; SERDP/ESTCP Environmental Remediation Technology; Springer: New York, 2010; pp 1–28.

[ref3] Stroo H. F.; Leeson A.; Marqusee J. A.; Johnson P. C.; Ward C. H.; Kavanaugh M. C.; Sale T. C.; Newell C. J.; Pennell K. D.; Lebrón C. A.; Unger M. Chlorinated Ethene Source Remediation: Lessons Learned. Environ. Sci. Technol. 2012, 46 (12), 6438–6447. 10.1021/es204714w. 22558915

[ref4] Bennett P.; He F.; Zhao D.; Aiken B.; Feldman L. In Situ Testing of Metallic Iron Nanoparticle Mobility and Reactivity in a Shallow Granular Aquifer. J. Contam. Hydrol. 2010, 116 (1–4), 35–46. 10.1016/j.jconhyd.2010.05.006. 20542350

[ref5] Kocur C. M.; Chowdhury A. I.; Sakulchaicharoen N.; Boparai H. K.; Weber K. P.; Sharma P.; Krol M. M.; Austrins L.; Peace C.; Sleep B. E.; O’Carroll D. M. Characterization of NZVI Mobility in a Field Scale Test. Environ. Sci. Technol. 2014, 48 (5), 2862–2869. 10.1021/es4044209. 24479900

[ref6] Zhang W.; Elliott D. W. Applications of Iron Nanoparticles for Groundwater Remediation. Remediat. J. 2006, 16 (2), 7–21. 10.1002/rem.20078.

[ref7] Bardos P.; Merly C.; Kvapil P.; Koschitzky H.-P. Status of Nanoremediation and Its Potential for Future Deployment: Risk-Benefit and Benchmarking Appraisals. Remediat. J. 2018, 28 (3), 43–56. 10.1002/rem.21559.

[ref8] Schöftner P.; Waldner G.; Lottermoser W.; Stöger-Pollach M.; Freitag P.; Reichenauer T. G. Electron Efficiency of NZVI Does Not Change with Variation of Environmental Parameters. Sci. Total Environ. 2015, 535, 69–78. 10.1016/j.scitotenv.2015.05.033. 26006053

[ref9] Reinsch B. C.; Forsberg B.; Penn R. L.; Kim C. S.; Lowry G. V. Chemical Transformations during Aging of Zerovalent Iron Nanoparticles in the Presence of Common Groundwater Dissolved Constituents. Environ. Sci. Technol. 2010, 44 (9), 3455–3461. 10.1021/es902924h. 20380376

[ref10] Liu H.; Wang Q.; Wang C.; Li X. Electron Efficiency of Zero-Valent Iron for Groundwater Remediation and Wastewater Treatment. Chem. Eng. J. 2013, 215–216, 90–95. 10.1016/j.cej.2012.11.010.

[ref11] Micić V.; Bossa N.; Schmid D.; Wiesner M. R.; Hofmann T. Groundwater Chemistry Has a Greater Influence on the Mobility of Nanoparticles Used for Remediation than the Chemical Heterogeneity of Aquifer Media. Environ. Sci. Technol. 2020, 54 (2), 1250–1257. 10.1021/acs.est.9b06135. 31860289

[ref12] Micić V.; Schmid D.; Bossa N.; Gondikas A.; Velimirovic M.; von der Kammer F.; Wiesner M. R.; Hofmann T. Impact of Sodium Humate Coating on Collector Surfaces on Deposition of Polymer-Coated Nanoiron Particles. Environ. Sci. Technol. 2017, 51 (16), 9202–9209. 10.1021/acs.est.7b01224. 28682625 PMC5802353

[ref13] Phenrat T.; Saleh N.; Sirk K.; Tilton R. D.; Lowry G. V. Aggregation and Sedimentation of Aqueous Nanoscale Zerovalent Iron Dispersions. Environ. Sci. Technol. 2007, 41 (1), 284–290. 10.1021/es061349a. 17265960

[ref14] Mueller N. C.; Braun J.; Bruns J.; Černík M.; Rissing P.; Rickerby D.; Nowack B. Application of Nanoscale Zero Valent Iron (NZVI) for Groundwater Remediation in Europe. Environ. Sci. Pollut. Res. 2012, 19 (2), 550–558. 10.1007/s11356-011-0576-3. 21850484

[ref15] Keane E. Fate, Transport and Toxicity of Nanoscale Zero-Valent Iron (NZVI) Used during Superfund Remediation; Duke University: Durham, NC, 2009.

[ref16] Guan X.; Sun Y.; Qin H.; Li J.; Lo I. M. C.; He D.; Dong H. The Limitations of Applying Zero-Valent Iron Technology in Contaminants Sequestration and the Corresponding Countermeasures: The Development in Zero-Valent Iron Technology in the Last Two Decades (1994–2014). Water Res. 2015, 75, 224–248. 10.1016/j.watres.2015.02.034. 25770444

[ref17] Stefaniuk M.; Oleszczuk P.; Ok Y. S. Review on Nano Zerovalent Iron (NZVI): From Synthesis to Environmental Applications. Chem. Eng. J. 2016, 287, 618–632. 10.1016/j.cej.2015.11.046.

[ref18] Fan D.; Lan Y.; Tratnyek P. G.; Johnson R. L.; Filip J.; O’Carroll D. M.; Nunez Garcia A.; Agrawal A. Sulfidation of Iron-Based Materials: A Review of Processes and Implications for Water Treatment and Remediation. Environ. Sci. Technol. 2017, 51, 13070 10.1021/acs.est.7b04177. 29035566

[ref19] Yan W.; Herzing A. A.; Li X.; Kiely C. J.; Zhang W. Structural Evolution of Pd-Doped Nanoscale Zero-Valent Iron (NZVI) in Aqueous Media and Implications for Particle Aging and Reactivity. Environ. Sci. Technol. 2010, 44 (11), 4288–4294. 10.1021/es100051q. 20446741

[ref20] Liu W.-J.; Qian T.-T.; Jiang H. Bimetallic Fe Nanoparticles: Recent Advances in Synthesis and Application in Catalytic Elimination of Environmental Pollutants. Chem. Eng. J. 2014, 236, 448–463. 10.1016/j.cej.2013.10.062.

[ref21] Kunze J. Nitrogen and Carbon in Iron and Steel Thermodynamics (Physical Research); Akademie Verlag: Berlin, 1990.

[ref22] Fry A. Stickstoff in Eisen, Stahl Und Sonderstahl. Ein Neues Oberflachenhartungsverfahren. Stahl und Eisen 1923, 43, 1271.

[ref23] Bhattacharyya S. Iron Nitride Family at Reduced Dimensions: A Review of Their Synthesis Protocols and Structural and Magnetic Properties. J. Phys. Chem. C 2015, 119 (4), 1601–1622. 10.1021/jp510606z.

[ref24] Yasavol N.; Mahboubi F. The Effect of Duplex Plasma Nitriding-Oxidizing Treatment on the Corrosion Resistance of AISI 4130 Steel. Mater. Des. 2012, 38, 59–63. 10.1016/j.matdes.2012.01.047.

[ref25] Moszyński D. Nitriding of Nanocrystalline Iron in the Atmospheres with Variable Nitriding Potential. J. Phys. Chem. C 2014, 118 (28), 15440–15447. 10.1021/jp500349d.

[ref26] Arabczyk W.; Zamłynny J.; Moszyński D. Kinetics of Nanocrystalline Iron Nitriding. Polish J. Chem. Technol. 2010, 12 (1), 38–43. 10.2478/v10026-010-0008-z.

[ref27] Mittemeijer E. J.; Slycke J. T. Chemical potentials and activities of nitrogen and carbon imposed by gaseous nitriding and carburising atmospheres. Surf. Eng. 1996, 12 (2), 152–162. 10.1179/sur.1996.12.2.152.

[ref28] Mittemeijer E. J.; Somers M. A. J. Thermodynamics, Kinetics, and Process Control of Nitriding. Surf. Eng. 1997, 13 (6), 483–497. 10.1179/sur.1997.13.6.483.

[ref29] Bouanis F. Z.; Bentiss F.; Traisnel M.; Jama C. Enhanced Corrosion Resistance Properties of Radiofrequency Cold Plasma Nitrided Carbon Steel: Gravimetric and Electrochemical Results. Electrochim. Acta 2009, 54 (8), 2371–2378. 10.1016/j.electacta.2008.10.068.

[ref30] Bouanis F. Z.; Jama C.; Traisnel M.; Bentiss F. Study of Corrosion Resistance Properties of Nitrided Carbon Steel Using Radiofrequency N2/H2 Cold Plasma Process. Corros. Sci. 2010, 52 (10), 3180–3190. 10.1016/j.corsci.2010.05.021.

[ref31] Cocke D. L.; Jurčik-Rajman M.; Vepřek S. The Surface Properties and Reactivities of Plasma-Nitrided Iron and Their Relation to Corrosion Passivation. J. Electrochem. Soc. 1989, 136 (12), 3655–3662. 10.1149/1.2096526.

[ref32] Weber T.; de Wit L.; Saris F. W.; Königer A.; Rauschenbach B.; Wolf G. K.; Krauss S. Hardness and Corrosion Resistance of Single-Phase Nitride and Carbide on Iron. Mater. Sci. Eng., A 1995, 199 (2), 205–210. 10.1016/0921-5093(94)09729-1.

[ref33] Anderson R. B. Nitrided Iron Catalysts for the Fischer–Tropsch Synthesis in the Eighties. Catal. Rev. 1980, 21 (1), 53–71. 10.1080/03602458008068060.

[ref34] Zheng M.; Chen X.; Cheng R.; Li N.; Sun J.; Wang X.; Zhang T. Catalytic Decomposition of Hydrazine on Iron Nitride Catalysts. Catal. Commun. 2006, 7 (3), 187–191. 10.1016/j.catcom.2005.10.009.

[ref35] Pelka R.; Moszyńska I.; Arabczyk W. Catalytic Ammonia Decomposition Over Fe/Fe4N. Catal. Lett. 2009, 128 (1–2), 72–76. 10.1007/s10562-008-9758-0.

[ref36] Wang L.; Xin Q.; Zhao Y.; Zhang G.; Dong J.; Gong W.; Guo H. In Situ FT-IR Studies on Catalytic Nature of Iron Nitride: Identification of the N Active Site. ChemCatChem. 2012, 4 (5), 624–627. 10.1002/cctc.201100311.

[ref37] Wang J.; Wang C.; Tong S. A Novel Composite Fe-N/O Catalyst for the Effective Enhancement of Oxidative Capacity of Persulfate at Ambient Temperature. Catal. Commun. 2018, 103, 105–109. 10.1016/j.catcom.2017.09.029.

[ref38] Yu F.; Zhou H.; Zhu Z.; Sun J.; He R.; Bao J.; Chen S.; Ren Z. Three-Dimensional Nanoporous Iron Nitride Film as an Efficient Electrocatalyst for Water Oxidation. ACS Catal. 2017, 7 (3), 2052–2057. 10.1021/acscatal.6b03132.

[ref39] Li J.; Yu F.; Wang M.; Lai Y.; Wang H.; Lei X.; Fang J. Highly Dispersed Iron Nitride Nanoparticles Embedded in N Doped Carbon as a High Performance Electrocatalyst for Oxygen Reduction Reaction. Int. J. Hydrogen Energy 2017, 42 (5), 2996–3005. 10.1016/j.ijhydene.2016.12.148.

[ref40] Wang M.; Yang Y.; Liu X.; Pu Z.; Kou Z.; Zhu P.; Mu S. The Role of Iron Nitrides in the Fe-N-C Catalysis System towards the Oxygen Reduction Reaction. Nanoscale 2017, 9 (22), 7641–7649. 10.1039/C7NR01925D. 28540947

[ref41] Zhang F.; Xi S.; Lin G.; Hu X.; Lou X. W. D.; Xie K. Metallic Porous Iron Nitride and Tantalum Nitride Single Crystals with Enhanced Electrocatalysis Performance. Adv. Mater. 2019, 31 (7), 1806552 10.1002/adma.201806552. 30575143

[ref42] Gong L.; Qiu X.; Tratnyek P. G.; Liu C.; He F. FeN X (C)-Coated Microscale Zero-Valent Iron for Fast and Stable Trichloroethylene Dechlorination in Both Acidic and Basic PH Conditions. Environ. Sci. Technol. 2021, 55 (8), 5393–5402. 10.1021/acs.est.0c08176. 33729752

[ref43] Kašlík J.; Kolařík J.; Filip J.; Medřík I.; Tomanec O.; Petr M.; Malina O.; Zbořil R.; Tratnyek P. G. Nanoarchitecture of Advanced Core-Shell Zero-Valent Iron Particles with Controlled Reactivity for Contaminant Removal. Chem. Eng. J. 2018, 354, 335–345. 10.1016/j.cej.2018.08.015.

[ref44] US EPA. Methods for Measuring the Acute Toxicity of Effluents and Receiving Waters to Freshwater and Marine Organisms, 5th ed.; Washington, DC, 2002.

[ref45] Brumovský M.; Filip J.; Malina O.; Oborná J.; Sracek O.; Reichenauer T. G.; Andrýsková P.; Zbořil R. Core-Shell Fe/FeS Nanoparticles with Controlled Shell Thickness for Enhanced Trichloroethylene Removal. ACS Appl. Mater. Interfaces 2020, 12 (31), 35424–35434. 10.1021/acsami.0c08626. 32640155 PMC7404211

[ref46] Kuhnen C. A.; de Figueiredo R. S.; Drago V.; da Silva E. Z. Mössbauer Studies and Electronic Structure of Γ•-Fe4N. J. Magn. Magn. Mater. 1992, 111 (1–2), 95–104. 10.1016/0304-8853(92)91062-X.

[ref47] Kurian S.; Gajbhiye N. S. Mössbauer and Magnetic Studies of Nanocrystalline γ′-Fe4N. In ICAME 2007; Gajbhiye N. S., Date S. K.; Springer: Berlin, Heidelberg, 2008; pp 319–325.

[ref48] Gorski C. A.; Scherer M. M. Determination of Nanoparticulate Magnetite Stoichiometry by Mossbauer Spectroscopy, Acidic Dissolution, and Powder X-Ray Diffraction: A Critical Review. Am. Mineral. 2010, 95 (7), 1017–1026. 10.2138/am.2010.3435.

[ref49] Kurian S.; Gajbhiye N. S. Magnetic and Mössbauer Study of δ-Fe y N (2 < *y* < 3) Nanoparticles. J. Nanoparticle Res. 2010, 12 (4), 1197–1209. 10.1007/s11051-009-9793-9.

[ref50] Ningthoujam R. S.; Gajbhiye N. S. Magnetic Study of Single Domain ε-Fe3N Nanoparticles Synthesized by Precursor Technique. Mater. Res. Bull. 2008, 43 (5), 1079–1085. 10.1016/j.materresbull.2007.06.011.

[ref51] Panda R. N.; Gajbhiye N. S. Magnetic Properties of Single Domain δ-Fe 3 N Synthesized by Borohydride Reduction Route. J. Appl. Phys. 1997, 81 (1), 335–339. 10.1063/1.364115.

[ref52] Bhattacharyya S.; Shivaprasad S. M.; Gajbhiye N. S. Variation of Magnetic Ordering in δ-Fe3N Nanoparticles. Chem. Phys. Lett. 2010, 496 (1–3), 122–127. 10.1016/j.cplett.2010.07.030.

[ref53] Sato S.; Omori K.; Araki S.; Takahashi Y.; Wagatsuma K. Surface Analysis of Nitride Layers Formed on Fe-Based Alloys through Plasma Nitride Process. Surf. Interface Anal. 2009, 41 (6), 496–501. 10.1002/sia.3053.

[ref54] Li D.; Choi C. J.; Kim B. K.; Zhang Z. D. Characterization of Fe/N Nanoparticles Synthesized by the Chemical Vapor Condensation Process. J. Magn. Magn. Mater. 2004, 277 (1–2), 64–70. 10.1016/j.jmmm.2003.10.011.

[ref55] Gontijo L. C.; Machado R.; Miola E. J.; Casteletti L. C.; Nascente P. A. P. Characterization of Plasma-Nitrided Iron by XRD, SEM and XPS. Surf. Coat. Technol. 2004, 183 (1), 10–17. 10.1016/j.surfcoat.2003.06.026.

[ref56] Sifkovits M.; Smolinski H.; Hellwig S.; Weber W. Interplay of Chemical Bonding and Magnetism in Fe4N, Fe3N and ζ-Fe2N. J. Magn. Magn. Mater. 1999, 204 (3), 191–198. 10.1016/S0304-8853(99)00296-6.

[ref57] Rohith Vinod K.; Saravanan P.; Sakar M.; Balakumar S. Insights into the Nitridation of Zero-Valent Iron Nanoparticles for the Facile Synthesis of Iron Nitride Nanoparticles. RSC Adv. 2016, 6 (51), 45850–45857. 10.1039/C6RA04935D.

[ref58] Wang X.; Zheng W. T.; Tian H. W.; Yu S. S.; Wang L. L. Effect of Substrate Temperature and Bias Voltage on DC Magnetron Sputtered Fe-N Thin Films. J. Magn. Magn. Mater. 2004, 283 (2–3), 282–290. 10.1016/j.jmmm.2004.06.002.

[ref59] Xu J.; Wang Y.; Weng C.; Bai W.; Jiao Y.; Kaegi R.; Lowry G. V. Reactivity, Selectivity, and Long-Term Performance of Sulfidized Nanoscale Zerovalent Iron with Different Properties. Environ. Sci. Technol. 2019, 53 (10), 5936–5945. 10.1021/acs.est.9b00511. 31022346

[ref60] Roh Y.; Lee S. Y.; Elless M. P. Characterization of Corrosion Products in the Permeable Reactive Barriers. Environ. Geol. 2000, 40 (1–2), 184–194. 10.1007/s002540000178.

[ref61] Génin J.-M. R.; Christy A.; Kuzmann E.; Mills S.; Ruby C. Structure and Occurrences of ≪ Green Rust ≫ Related New Minerals of the ≪ Fougérite ≫ Group, Trébeurdenite and Mössbauerite, Belonging to the ≪ Hydrotalcite ≫ Supergroup; How Mössbauer Spectroscopy Helps XRD. Hyperfine Interact. 2014, 226 (1–3), 459–482. 10.1007/s10751-014-1045-4.

[ref62] Hwang Y.; Shin H.-S. Effects on Nano Zero-Valent Iron Reactivity of Interactions between Hardness, Alkalinity, and Natural Organic Matter in Reverse Osmosis Concentrate. J. Environ. Sci. 2013, 25 (11), 2177–2184. 10.1016/S1001-0742(12)60323-4. 24552045

[ref63] Bae S.; Collins R. N.; Waite T. D.; Hanna K. Advances in Surface Passivation of Nanoscale Zerovalent Iron: A Critical Review. Environ. Sci. Technol. 2018, 52 (21), 12010–12025. 10.1021/acs.est.8b01734. 30277777

[ref64] Kaya D.; Kjellerup B. V.; Chourey K.; Hettich R. L.; Taggart D. M.; Löffler F. E. Impact of Fixed Nitrogen Availability on Dehalococcoides Mccartyi Reductive Dechlorination Activity. Environ. Sci. Technol. 2019, 53 (24), 14548–14558. 10.1021/acs.est.9b04463. 31693350

[ref65] He F.; Li Z.; Shi S.; Xu W.; Sheng H.; Gu Y.; Jiang Y.; Xi B. Dechlorination of Excess Trichloroethene by Bimetallic and Sulfidated Nanoscale Zero-Valent Iron. Environ. Sci. Technol. 2018, 52 (15), 8627–8637. 10.1021/acs.est.8b01735. 29952547

[ref66] Fan D.; O’Carroll D. M.; Elliott D. W.; Xiong Z.; Tratnyek P. G.; Johnson R. L.; Garcia A. N. Selectivity of Nano Zerovalent Iron in In Situ Chemical Reduction: Challenges and Improvements. Remediat. J. 2016, 26 (4), 27–40. 10.1002/rem.21481.

[ref67] Mangayayam M. C.; Perez J. P. H.; Dideriksen K.; Freeman H. M.; Bovet N.; Benning L. G.; Tobler D. J. Structural Transformation of Sulfidized Zerovalent Iron and Its Impact on Long-Term Reactivity. Environ. Sci. Nano 2019, 6 (11), 3422–3430. 10.1039/C9EN00876D.

[ref68] Schöftner P.; Waldner G.; Lottermoser W.; Stöger-Pollach M.; Freitag P.; Reichenauer T. G. Electron Efficiency of NZVI Does Not Change with Variation of Environmental Parameters. Sci. Total Environ. 2015, 535, 69–78. 10.1016/j.scitotenv.2015.05.033. 26006053

[ref69] Arnold W. A.; Roberts A. L. Pathways and Kinetics of Chlorinated Ethylene and Chlorinated Acetylene Reaction with Fe(0) Particles. Environ. Sci. Technol. 2000, 34 (9), 1794–1805. 10.1021/es990884q.

[ref70] Liu Y.; Majetich S. A.; Tilton R. D.; Sholl D. S.; Lowry G. V. TCE Dechlorination Rates, Pathways, and Efficiency of Nanoscale Iron Particles with Different Properties. Environ. Sci. Technol. 2005, 39 (5), 1338–1345. 10.1021/es049195r. 15787375

[ref71] Deng B.; Campbell T. J.; Burris D. R. Hydrocarbon Formation in Metallic Iron/Water Systems. Environ. Sci. Technol. 1997, 31 (4), 1185–1190. 10.1021/es960698+.

[ref72] Schulz H. Short History and Present Trends of Fischer–Tropsch Synthesis. Appl. Catal. A Gen. 1999, 186 (1–2), 3–12. 10.1016/S0926-860X(99)00160-X.

[ref73] Liu Y.; Phenrat T.; Lowry G. V. Effect of TCE Concentration and Dissolved Groundwater Solutes on NZVI-Promoted TCE Dechlorination and H 2 Evolution. Environ. Sci. Technol. 2007, 41 (22), 7881–7887. 10.1021/es0711967. 18075103

[ref74] Letchworth-Weaver K.; Arias T. A. Joint Density Functional Theory of the Electrode-Electrolyte Interface: Application to Fixed Electrode Potentials, Interfacial Capacitances, and Potentials of Zero Charge. Phys. Rev. B 2012, 86 (7), 075140 10.1103/PhysRevB.86.075140.

[ref75] Mathew K.; Sundararaman R.; Letchworth-Weaver K.; Arias T. A.; Hennig R. G. Implicit Solvation Model for Density-Functional Study of Nanocrystal Surfaces and Reaction Pathways. J. Chem. Phys. 2014, 140 (8), 084106 10.1063/1.4865107. 24588147

[ref76] Shi Y. J.; Du Y. L.; Chen G. Ab Initio Study of Structural and Magnetic Properties of Cubic Fe4N(001) Surface. Solid State Commun. 2012, 152 (16), 1581–1584. 10.1016/j.ssc.2012.05.017.

[ref77] Lim D.-H.; Lastoskie C. M.; Soon A.; Becker U. Density Functional Theory Studies of Chloroethene Adsorption on Zerovalent Iron. Environ. Sci. Technol. 2009, 43 (4), 1192–1198. 10.1021/es802523a. 19320179

[ref78] Lim D.-H.; Lastoskie C. M. Density Functional Theory Studies on the Relative Reactivity of Chloroethenes on Zerovalent Iron. Environ. Sci. Technol. 2009, 43 (14), 5443–5448. 10.1021/es9003203. 19708379

[ref79] Kolos M.; Tunega D.; Karlický F. A Theoretical Study of Adsorption on Iron Sulfides towards Nanoparticle Modeling. Phys. Chem. Chem. Phys. 2020, 22 (40), 23258–23267. 10.1039/D0CP02988B. 33030174

[ref80] Zhang N.; Luo J.; Blowers P.; Farrell J. Understanding Trichloroethylene Chemisorption to Iron Surfaces Using Density Functional Theory. Environ. Sci. Technol. 2008, 42 (6), 2015–2020. 10.1021/es0717663. 18409630 PMC3700525

[ref81] Filip J.; Karlický F.; Marušák Z.; Lazar P.; Černík M.; Otyepka M.; Zbořil R. Anaerobic Reaction of Nanoscale Zerovalent Iron with Water: Mechanism and Kinetics. J. Phys. Chem. C 2014, 118 (25), 13817–13825. 10.1021/jp501846f.

[ref82] Li H.; Yang W.; Wu C.; Xu J. Origin of the Hydrophobicity of Sulfur-Containing Iron Surfaces. Phys. Chem. Chem. Phys. 2021, 23 (25), 13971–13976. 10.1039/D1CP00588J. 34143174

[ref83] Schneidewind U.; Haest P. J.; Atashgahi S.; Maphosa F.; Hamonts K.; Maesen M.; Calderer M.; Seuntjens P.; Smidt H.; Springael D.; Dejonghe W. Kinetics of Dechlorination by Dehalococcoides Mccartyi Using Different Carbon Sources. J. Contam. Hydrol. 2014, 157, 25–36. 10.1016/j.jconhyd.2013.10.006. 24275111

