# Supplementary material for: Iron Nitride Nanoparticles for Enhanced Reductive Dechlorination of Trichloroethylene
Source: Environ Sci Technol. 2022 Mar 9;56(7):4425–36. doi: 10.1021/acs.est.1c08282 (PMC8988298; doi:10.1021/acs.est.1c08282)
Supplement: Supplementary file 1 — es1c08282_si_001.pdf [file es1c08282_si_001.pdf]

Supporting Information

Iron Nitride Nanoparticles for Enhanced Reductive Dechlorination of Trichloroethylene

*Miroslav Brumovský [a, b, c, *], Jana Oborná [b], Vesna Micić [a], Ondřej Malina [b], Josef Kašlík [b], Daniel Tunega [c, d], Miroslav Kolos [e], Thilo Hofmann [a], František Karlický [e], Jan Filip [b]*

[a] Department of Environmental Geosciences (EDGE), Centre for Microbiology and Environmental Systems Science, University of Vienna, Althanstr. 14, UZA II, 1090 Vienna, Austria

[b] Regional Centre of Advanced Technologies and Materials, Czech Advanced Technology and Research Institute (CATRIN), Palacký University Olomouc, Šlechtitelů 27, 779 00 Olomouc, Czech Republic

[c] University of Natural Resources and Life Sciences, Vienna, Department of Forest- and Soil Sciences, Institute of Soil Research, Peter-Jordan-Straße 82, 1190 Vienna, Austria

[d] School of Pharmaceutical Science and Technology, Tianjin University, 300072 Tianjin, P.R. China.

[e] Department of Physics, Faculty of Science, University of Ostrava, 701 03 Ostrava, Czech Republic

* Corresponding author at the Regional Centre of Advanced Technologies and Materials, Czech Advanced Technology and Research Institute (CATRIN), Palacký University Olomouc, Šlechtitelů 27, 779 00 Olomouc, Czech Republic
E-mail address: miroslav.brumovsky@upol.cz

Summary

Number of pages: 33

Number of tables: 16

Number of figures: 17

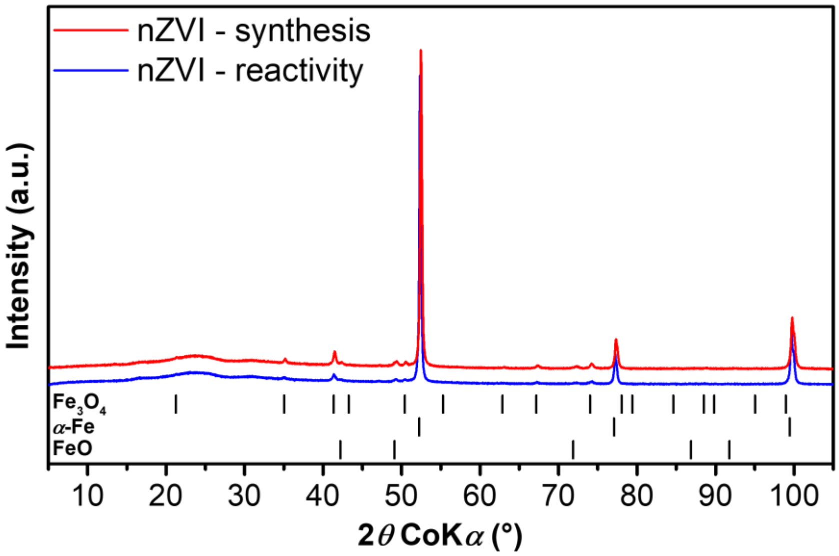

Figure S1. XRD patterns of nZVI particles (NANOFER 25P) used for the synthesis of Fe_xN particles (red) and used as reference material in aging and reactivity experiments (blue).

Table S1. Rietveld XRD quantification of Fe-containing mineral phases in the XRD profiles of fresh nZVI particles (NANOFER 25P) used in this study.

Particle type	Abundance of crystalline phases (wt. %)		
	***α*-Fe**	**Fe_3O_4**	**FeO**
nZVI used for the synthesis of Fe_xN particles	87.2	8.9	3.9
nZVI used as reference material in aging and reactivity experiments	93.8	4.4	1.8

Text S1. Chemicals and Materials

Liquid chlorinated ethenes trichloroethylene (≥99.9%), 1,1-dichloroethene (98.5%), *trans*-1,2-dichloroethene (98%), and *cis*-1,2-dichloroethene (≥95%, GC grade) were purchased from Sigma-Aldrich (USA). Gases were purchased from Linde AG (Austria): H_2 (5.0), Ar (5.0), N_2 (5.0), methane (60% ± 1% in CO_2), ethene (997 ppm (v/v) ± 2% in N_2), acetylene (985 ppm (v/v) ± 2% in N_2), ethane (992 ppm (v/v) ± 2% in N_2), vinyl chloride (1047 ppm (v/v) ± 2% in N_2), hydrogen (1% ± 2% (v/v) in N_2), synthetic air (HC free, 20% O_2 in N_2), propane (60%), and a multi-component standard (methane

1007 ppm (v/v) ± 2%, ethene 993 ppm (v/v) ± 2%, ethane 976 ppm (V/V) ± 2%, ethane 1013 ppm (v/v) ± 2%, vinyl chloride 1032 ppm (v/v) ± 2%, and trichloroethylene 2438 ppm (v/v) ± 2% in N_2). Hydrochloric acid (37%), sodium hydroxide, methanol (≥ 99.8%), and ethanol (≥99.5%) were purchased from Merck (Germany). 20x sodium bicarbonate/sodium carbonate concentrate (Sigma-Aldrich, USA) and a Merck Certipur Anion multi-element standard II (1 g/L Cl^-, NO_3^-, SO_4^{2-}) were used for ion chromatography. Iron standard for AAS (1 g/L) was purchased from Fluka Analytical (Sigma-Aldrich, Germany). Ultrapure helium (99.99990%) used as the carrier gas in the non-target analysis, and anhydrous ammonia (99.98%) and nitrogen (99.999%) used for nitriding were obtained from Messer Technogas s. r. o. (Czech Republic). Sodium sulfide nonahydrate (≥98.0%) used for the synthesis of S-nZVI was purchased from Alfa Aesar, Germany.

Table S2. Composition and physico-chemical parameters of synthetic moderately hard water (MHW) prepared according to the U.S. EPA protocol[1] and sparged with N_2 for 45 min. The concentrations of cations were determined by ICP-OES (Optima 5300 DV, PerkinElmer). The concentrations of sulfate and chloride were determined by IC (930 Compact IC Flex, Metrohm). The concentration of HCO_3^- was determined by titration with 0.1 N HCl to pH 4.3.[2] The pH was measured with a pre-calibrated combination electrode (SenTix® 41, WTW). Conductivity was measured using a four-electrode-conductivity cell (TetraCon® 325, WTW).

Ion concentration (mmol L^{-1})							pH	Conductivity (µS cm^{-1})	Ionic strength* (mmol L^{-1})
Na^+	K^+	Ca^{2+}	Mg^{2+}	HCO_3^-	SO_4^{2-}	Cl^-			
1.00	0.069	0.35	0.48	1.14	0.82	0.055	8.2	300	4.8

* Calculated as IS (mmol L^{-1}) = 1.6×10^{-5} × conductivity (µS cm^{-1}).[3]

Table S3. Nitriding experimental conditions.

Particle type	Composition of the nitriding gas (NH_3:N_2)	Gas flow (L h^{-1})	Temperature (°C)	Time (h)
γ'-Fe_xN	1:2	30	500	3
ε-Fe_xN	2:1	30	300	5.5

Text S2. Particle Characterization

Sample Preparation

Fresh particles were surface passivated before characterization. The surface passivation consisted of particle suspension in water using the same procedure as used for aging and reactivity experiments, i.e., 4 g of particles were stirred with 16 mL of deoxygenated ultrapure water using T25 ULTRA-TURRAX® disperser (IKA, Germany) at 11 000 rpm for 2 min. Subsequently, the particles were separated with a magnet, the supernatant was discarded, the remaining pellet was either washed with ethanol three times and dried in an Ar-filled glovebox for 48 h (samples for water contact angle measurements, BET, and elemental analysis) or flash-frozen in liquid N_2 (samples for other characterization methods). Samples were stored either in airtight tubes filled with an inert atmosphere inside the glovebox or in a freezer at –80 °C before analysis, respectively. Aged particles were separated from suspensions with a magnet, the supernatant was discarded, and the remaining pellet was flash-frozen in liquid N_2 (at –196 °C). Samples were stored at –80 °C before analysis.

Powder X-Ray Diffraction

The phase composition of fresh and aged particles was determined by X-ray powder diffraction (XRD) with an Aeris diffractometer (PANalytical, B.V.) operating in Bragg-Brentano geometry. Defrozen samples were transferred on a zero-diffraction silicone sample holder and immediately covered with Kapton foil to protect the samples against degradation. The diffractometer was equipped with a CoKα radiation source, fixed divergence, diffracted beam anti-scatter slits, and PIXcel detector. The patterns were measured in the 2θ range from 5 to 105° and the data were processed using HighScorePlus software in conjunction with PDF-4+ and ICSD databases. Crystalline phases were quantified using the Rietveld refinement.[4]

Mössbauer Spectroscopy

Iron speciation was analyzed using a low-temperature (–123 °C) Mössbauer spectrometer on isotope ^{57}Fe (MS2007 instrument), operating in a constant acceleration mode and equipped with a 50 mCi ^{57}Co(Rh) source.[5,6] Defrozen samples were transferred to a plastic in-house-built sample cuvette, immediately frozen in liquid N_2 (-196 °C), and mounted in a cooling chamber (-123 °C) where the samples were analyzed. MossWinn software[7] was used for fitting the Mössbauer spectra. The values of the isomer shift were referred to an α-Fe foil sample at room temperature. The effects of non-ideal absorber thickness and variable recoil-free fractions for iron atoms in non-equivalent structural sites of

different phases were expected to be within experimental error (hyperfine parameters ±0.02 mm s^{-1}, relative spectral area ±2%).

X-Ray Photoelectron Spectroscopy

Chemical states of major elements and their distribution on the particle surface were evaluated by X-ray photoelectron spectroscopy (XPS) with a PHI 5000 VersaProbe II XPS system (Physical Electronics, Inc., MN, USA) using monochromatic AlKα radiation (15 kV, 50 W) and photon energy of 1486.7 eV. Defrozen samples were mounted on a sample holder by double-sided tape (SCOTCH) and immediately inserted in a vacuum chamber where the sample was dried and subsequently analyzed. The survey spectra were measured with a pass energy of 187.85 eV and a step of 0.8 eV while for the high-resolution spectra pass energy of 23.5 eV and a step of 0.2 eV were used. Dual beam charge compensation was used for all measurements. All the spectra were measured in the vacuum of 1.4 x 10^{-7} Pa at room temperature (20 °C). The analyzed area on each sample was 100 μm in diameter. The spectra were evaluated with the MultiPak (Ulvac - PHI, Inc.) software. All binding energy values were referenced to the aliphatic carbon peak C1s at 284.80 eV.

Fe, Fe^0, and N Content

The total Fe content in the Fe_xN particles was determined on an HNO_3-digested sample using electrothermal atomic absorption spectrometry (AAS) with a graphite furnace (ContrAA 600, Analytik Jena AG, Germany) equipped with a high-resolution Echelle double monochromator (spectral bandwidth, 2 pm at 200 nm). A xenon lamp was used as a continuum radiation source and an absorption line at 248.3 nm was used for quantification. The total N content was determined on a dry and homogenized sample using an Elemental Vario MACRO CHNS analyzer (Germany), with a laboratory-determined relative standard deviation of ± 0.8%. Before the analysis, the instrument calibration was adjusted to the daily ambient conditions using daily factor determination with the calibration standard sulfanilamide. The Fe^0 content in Fe_xN particles was determined using the volume of hydrogen produced after adding an excess of 1:1 mixture of HCl (37%) and ultrapure water to the particle suspensions. The Fe^0 content was calculated using the measured volume of the evolved hydrogen and the molar volume of an ideal gas at laboratory temperature. The reported values are the average value of three replicates.

Electron Microscopy

The particle morphology was examined by a combination of transmission electron microscopy (TEM) and scanning electron microscopy (SEM). For routine particle size/morphology observations, we used a TEM JEOL 2100 instrument equipped with X-MaxN 80T SDD EDS detector (Oxford Instruments) at an electron acceleration voltage of 200 kV and SEM Hitachi SU6600 (Hitachi, Japan) operating at 5 kV accelerating voltage. High-resolution TEM (HR-TEM) images of individual particles were recorded with spherical aberration-corrected Titan G2 (FEI) operated at 80 kV and a BM UltraScan CCD camera (Gatan). Energy Dispersive Spectrometry (EDS) was performed in the scanning (STEM) mode to obtain elemental maps by Super-X system with four silicon drift detectors (Bruker). STEM images were taken with HAADF detector 3000 (Fishione). The elemental mappings were performed on at least four representative images from different locations of the sample grid. Particle size and shell thickness distributions were evaluated using the ImageJ software (100 measurements were performed for each distribution). (HR)TEM and SEM imaging were conducted with thawed aqueous suspensions. Particles were diluted in ethanol, 1 drop of the diluted suspension was placed on a Holey-carbon coated 300 mesh Cu TEM grid and dried at room temperature.

Water Contact Angle Measurement

13 mm (diameter) by 2 mm (thick) pellets of both nitrided particle types were prepared using an H-62 laboratory hydraulic press (Trystom, Czech Republic). The pellets were dried in a vacuum oven at 60 °C for 8 h and then the pressure was gradually equalized with air over 10 minutes. The water contact angle of the vacuum-dried pellets was determined by axisymmetric drop shape analysis on a Krüss DSA 30 (Krüss GmbH, Germany) instrument. All measurements were performed with 2 μL of purified water (Millipore, USA) with a conductivity of 0.056 μS cm^{-1} and repeated 5× at 21 °C.

Brunauer-Emmett-Teller (BET) Specific Surface Area

The Brunauer-Emmett-Teller (BET) surface area was determined on dried samples using a NOVA 2000e Surface Area Analyzer (Quantachrome, U.S.A.) by multipoint BET analysis of the nitrogen adsorption data at the relative pressure of 0.05–0.3 at –196 °C. Before the measurements, all samples were outgassed at 25 °C for at least 24 h. The experimentally determined relative standard deviation was ± 2.8% and the average linear correlation coefficient of the BET plots was 0.9996. The reported BET surface area was an average value of three replicates.

Agglomerate Size Distribution

A 125 mg L^{-1} particle suspension in MHW was characterized by laser diffraction analysis (Mastersizer 2000, Malvern Instruments, U.K.) equipped with the Hydro 2000S dispersion unit. The speed of the pump and stirrer was set to 1 750 rpm and the ultrasonic level to 100%. Samples were analyzed in triplicate with an acquisition time of 60 s.

Dissolved Nitrogen-Containing Species

Dissolved ammonia ($NH_{3(aq)}$) and other nitrogen species (NO_2^- and NO_3^-) originating from Fe_xN particle leaching and HCl digestion were determined using distillation and titration method (ISO 5664) and liquid chromatography (ISO 10304-1), respectively, after separation of the solids with a magnet and filtration using 0.1 μm PTFE syringe filter.

Text S3. TCE Dechlorination Experiments

Batch experiments with fresh particles were conducted in 42-mL glass vials capped with PTFE-lined Mininert® valves (Sigma-Aldrich, Germany). The vials containing 20 mL of deoxygenated MHW were spiked with 82 μL of freshly prepared Fe_xN stock suspensions (20% w/w) to obtain a particle concentration of 1 g L^{-1}. Dechlorination experiments with aged particles were performed in serum bottles (see Section 2.3 in the manuscript). Reactivity experiments were started by injecting TCE stock solution in methanol (8 g L^{-1}) to obtain an initial TCE concentration of 20 mg L^{-1} (i.e., 50 μL for experiments with fresh particles and 150 μL for experiments with aged particles). The reaction vessels were then placed on a horizontal shaker (125 rpm) at 22 ± 1 °C. An aliquot (25–100 μL) of headspace gas was periodically withdrawn using a gastight syringe and analyzed using a 7890B gas chromatograph (GC, Agilent Technologies, USA) for the amount of TCE, its C_2-degradation products, and hydrogen for three weeks with a previously established method.[8] Control experiments with pristine nZVI were performed in parallel. A control experiment with fresh core-shell S-nZVI particles was also performed. The S-nZVI was synthesized according to our previous study with a S/Fe mass ratio of 0.94/100, which exhibited the highest electron efficiency and > 90% sulfur incorporation during synthesis.[8] Reactivity experiments with fresh and aged particles were done in four and two replicates, respectively. Measured amounts of analytes were compensated for overpressure and sampling-induced headspace losses as described elsewhere.[8,9] Total concentrations of all analytes,

observed pseudo-first-order reaction rate constants of TCE removal, k_{obs}, and initial surface-area normalized rate constant, k_{SA}, were calculated according to ref.[8].

Dissolved chloride (Cl^-) concentration at the end of degradation experiments was determined using the 930 IC Flex ion chromatography system (Metrohm, Switzerland).

Non-target headspace analysis was performed at the end of the dechlorination experiment with fresh γ'-Fe_xN nanoparticles to investigate the full range of TCE dechlorination products using a 7890B gas chromatograph (GC, Agilent Technologies, USA) coupled to a high-resolution time-of-flight (TOF) mass spectrometer 7250 Q-TOF (Agilent Technologies, USA). 100 µL of headspace gas was injected manually. The GC system was equipped with two columns HP-5MS 15 m x 250 µm x 0.25 µm (Agilent Technologies, USA). The injector temperature was set to 250 °C and a transfer line temperature to 280 °C. An oven temperature program (40 °C for 13.3 min, ramp 3.1 °C/min to 180 °C, then 11.5 °C/min to 250 °C and hold for 0.45 min) was applied. The carrier gas was ultrapure helium (99.99990%) set at the flow mode for the first column (1 mL/min) and second column (1.2 mL/min). The mass spectrometer was operated in the electron ionization mode at 70 eV. The TOF analyzer settings were as follows: the range of m/z was set from 20 to 300, the scan rate was set to 5 spectra/sec. The temperature of the ion source was set at 200 °C. The compound identification was performed in the Agilent MassHunter Unknown Analysis software (version B.09.00). Compounds with a match factor > 90 (on the normalized scale 0 – 100) with the NIST 17 mass spectral library were considered as tentatively identified dechlorination products.[10]

Text S4. Computational Methodology

Periodic spin-polarized density functional theory (DFT) calculations were performed in a plane-wave basis set using the Vienna ab initio Simulation Package (VASP).[11–14] Electronic structure of atoms was expressed by pseudopotentials using the projector-augmented wave (PAW) method.[15,16] Electronic exchange and correlation terms were described within the frame of the generalized gradient approximation (GGA) theory by using the Perdew-Burke-Ernzerhof (PBE) functional.[17] Dispersion correction energy was added to the PBE energy by employing the DFT-D3 dispersion correction scheme with the Becke-Jonson damping.[18] Kinetic energy cutoff for plane waves in all the calculations was set to 400 eV. A force convergence criterion for the ionic relaxation was set to 0.01 eV/Å, an

electronic step condition for the SCF convergence was 10^{-6} eV. Monkhorst-Pack 2 × 2 × 1 k-point grid scheme was used for Brillouin zone sampling.[19]

$Fe_{II}N$-terminated (001) surface of the γ'-Fe_4N slab was considered as a model for the iron nitride surface as it was found previously to be thermodynamically favorable.[20] The slab model was constructed from the fully relaxed γ'-Fe_4N bulk structure (including unit cell parameters) using the same setup as described above and 8 × 8 × 8 k-point scheme. The following physical properties were calculated for bulk γ'-Fe_4N: lattice constant 3.746 Å, Fe_I magnetic moment 2.9 μ_B and Fe_{II} magnetic moment 2.2 μ_B. These values are in good agreement with experimentally determined lattice constant 3.790 Å, Fe_I magnetic moment 3.0 μ_B, and Fe_{II} magnetic moment 2.0 μ_B.[21–24] The slab model consisted of a 4x4 supercell with three atomic planes in the direction parallel to the surface (two $Fe_{II}N$, top and bottom, and one Fe_I plane in between) being symmetric in the direction perpendicular to the surface and preserving artificial slab polarization. All atoms of the slab were allowed to relax keeping supercell parameters constant. To decouple adjacent slabs, a thick vacuum layer of 25 Å was included in the direction perpendicular to the surface. Gas-phase geometry optimization of the isolated TCE molecule was performed in a supercell of the same dimensions. To ensure the suitability of a three-layer γ'-Fe_4N model, control adsorption energy (E_{ads}) calculations of surface complexes were carried out with a seven-layer 4×4 supercell $Fe_{II}N$-terminated γ'-Fe_4N(001) model. The difference in E_{ads} was < 5 kJ/mol.

A surface slab model of α-Fe(110) with similar dimensions was constructed in the same fashion to allow for comparison between the reactivity of Fe_4N and Fe. The (110) surface was reported as the most stable α-Fe surface.[25] The model was prepared from the fully relaxed α-Fe bulk structure. The calculated lattice constant for bulk α-Fe was 2.812 Å and the Fe magnetic moment 2.2 μB. These values are in agreement with experimentally determined lattice constant 2.860 Å[26] and Fe magnetic moment 2.2 μB[27]. The slab model consisted of a 4x5 supercell with three atomic planes in the direction parallel to the surface with symmetric outer planes. A 4x4 supercell surface slab model of FeS(001) used for the calculation of water adsorption energy was prepared according to our previous study.[28]

The adsorption energies of TCE and H_2O were calculated as

$$E_{ads} = E_{complex} - (E_{slab} + E_{mol}), \tag{1}$$

where $E_{complex}$ is the total electronic energy of the relaxed complex between TCE/H_2O and the corresponding slab, and E_{slab} and E_{mol} are total electronic energies of the relaxed slab and the isolated TCE/H_2O molecule, respectively. The structure optimization of physisorbed TCE was performed for eight initial configurations on the γ'-Fe_4N slab and four initial configurations on the α-Fe slab (containing TCE in different orientations at ca. 5 Å above the slab) in two steps: first with the GADGET code[29] and subsequently with the conjugate-gradient algorithm as implemented in the VASP package. Only the physisorbed TCE-γ'-Fe_4N surface complex with the lowest energy is further discussed in this work. In all calculations, the full geometry relaxation of α-Fe surfaces with TCE led to spontaneous dechlorination of TCE to chloroacetylene. The structure of TCE chemisorbed onto the γ'-Fe_4N surface was optimized with the conjugate-gradient algorithm as implemented in the VASP package. The structure optimization of adsorbed H_2O was performed also in two steps as described above with four initial configurations for each slab. The effect of solvent on the TCE adsorption was included in the calculations by using an implicit solvation model developed for solid-liquid interfaces[30] and implemented to the VASP program (VASPsol).[31,32] This approach is similar to quantum mechanical polarizable continuum models developed for molecules in solvents.[33] The VASPsol calculations were performed as single-point calculations on the gas phase optimized geometries.

The energy barriers of the γ'-Fe_4N-mediated dechlorination of TCE were computed with the climbing image nudged elastic band (CI-NEB) method.[34] A total of seven intermediate images (excluding the initial reactant and final product structures) were used. Note that the calculation of energy barriers for the α-Fe-mediated TCE dechlorination was not possible due to spontaneous TCE dissociation during the geometry optimization, as discussed above. The transition states were verified by frequency analysis at the same computational level as used for geometry optimization, which showed exactly one imaginary frequency corresponding to the change in geometry along the reaction path.

The energy barrier ($E^{\ddagger}$) was calculated as relative to the energy of the reactant (E_{react})

$$E^{\ddagger} = E_{TS} - E_{react}, \quad (2)$$

where E_{TS} is the energy of the transition state. Computed structures were visualized using VESTA 3.[35]

The calculated homolytic bond dissociation energy (BDE) of the C–Cl bond in the isolated TCE molecule in the gas phase served as a reference energy barrier for the non-facilitated dechlorination reaction. To account for an artificial effect of the periodic boundaries on calculated BDE, we

performed these calculations with both VASP and the program TURBOMOLE[36] using the same PBE-D3 method and def2-TZVP basis set.[37,38] To compare the effect of solvent on the BDE energies, we performed also TURBOMOLE calculations involving a continuum solvation model, particularly the Conductor-like Screening Model (COSMO).[39] The results presented in Table S16 showed only a small difference between the BDEs calculated with VASP and TURBOMOLE and the same trend for different Cl atoms.

Table S4. Rietveld XRD quantification of Fe-containing mineral phases in the XRD profiles of fresh and three-month aged suspensions of Fe_xN and nZVI particles.

**Particle type**		**Abundance of crystalline phases (wt. %)**						
		**α-Fe**	**γ'-Fe_4N**	**ε-$Fe_{2\text{-}3}N$**	**Fe_3O_4**	**FeO**	**$MgCO_3$**	**Trébeurdenite**
Fresh	nZVI	93.8	--	--	4.4	1.8	--	--
	γ'-Fe_xN	--	90.7	9.3	--		--	--
	ε-Fe_xN	--	4.1	84.4	11.5		--	--
Aged 3 months	nZVI	1.1	--	--	4.0		4.2	90.7
	γ'-Fe_xN	--	40.1	--	--		--	59.9
	ε-Fe_xN	--	2.5	--	11.7		7.6	78.2

Table S5. Values of the Mössbauer hyperfine parameters, derived from the least-square fitting of the Mössbauer spectra of the fresh and three-month aged suspensions of γ'-Fe_xN and ε-Fe_xN nanoparticles, where δ is the isomer shift, ΔE_Q is the quadrupole splitting, B_{hf} is the hyperfine magnetic field, and RA is the relative spectral area of individual spectral components identified during fitting. The spectra were recorded at 150 K.

**Particle type**	**Component**	δ ± 0.01 (mm s^{-1})	ΔE_Q ± 0.01 (mm s^{-1})	B_{hf} ± 0.3 (T)	**RA ± 1 (%)**	**Assignment**
Fresh γ'-Fe_xN	Sextet 1	0.29	0.01	35.4	22	γ'-Fe_4N (Fe^C)
	Sextet 2	0.36	0.01	22.5	65	γ'-Fe_4N (Fe^f-I)
	Sextet 3	0.30	-0.40	21.7	5	γ'-Fe_4N (Fe^f-II)
	Sextet 4	0.40	-0.02	23.4	8	ε-Fe_3N
Fresh ε-Fe_xN	Sextet 1	0.25	0.00	48.0	10	Fe_3O_4 - T-sites
	Sextet 2	0.69	0.00	46.0	19	Fe_3O_4 - O-sites
	Doublet	0.47	0.12	--	71	ε-Fe_3N
Aged γ'-Fe_xN	Sextet 1	0.29	0.01	35.4	8	γ'-Fe_4N (Fe^C)
	Sextet 2	0.36	0.01	22.5	15	γ'-Fe_4N (Fe^f-I)
	Sextet 3	0.30	-0.40	21.7	5	γ'-Fe_4N (Fe^f-II)
	Doublet 1	1.24	2.69	--	59	Trébeurdenite (Fe^{2+})
	Doublet 2	0.25	0.79	--	13	Trébeurdenite (Fe^{3+})
Aged ε-Fe_xN	Sextet 1	0.44	0.00	49.2	5	Fe_3O_4 - T-sites
	Sextet 2	0.75	0.00	46.8	6	Fe_3O_4 - O-sites
	Doublet 1	1.23	2.73	--	69	Trébeurdenite (Fe^{2+})
	Doublet 2	0.20	0.82	--	15	Trébeurdenite (Fe^{3+})
	Doublet 3	0.47	0.12	--	5	ε-Fe_3N

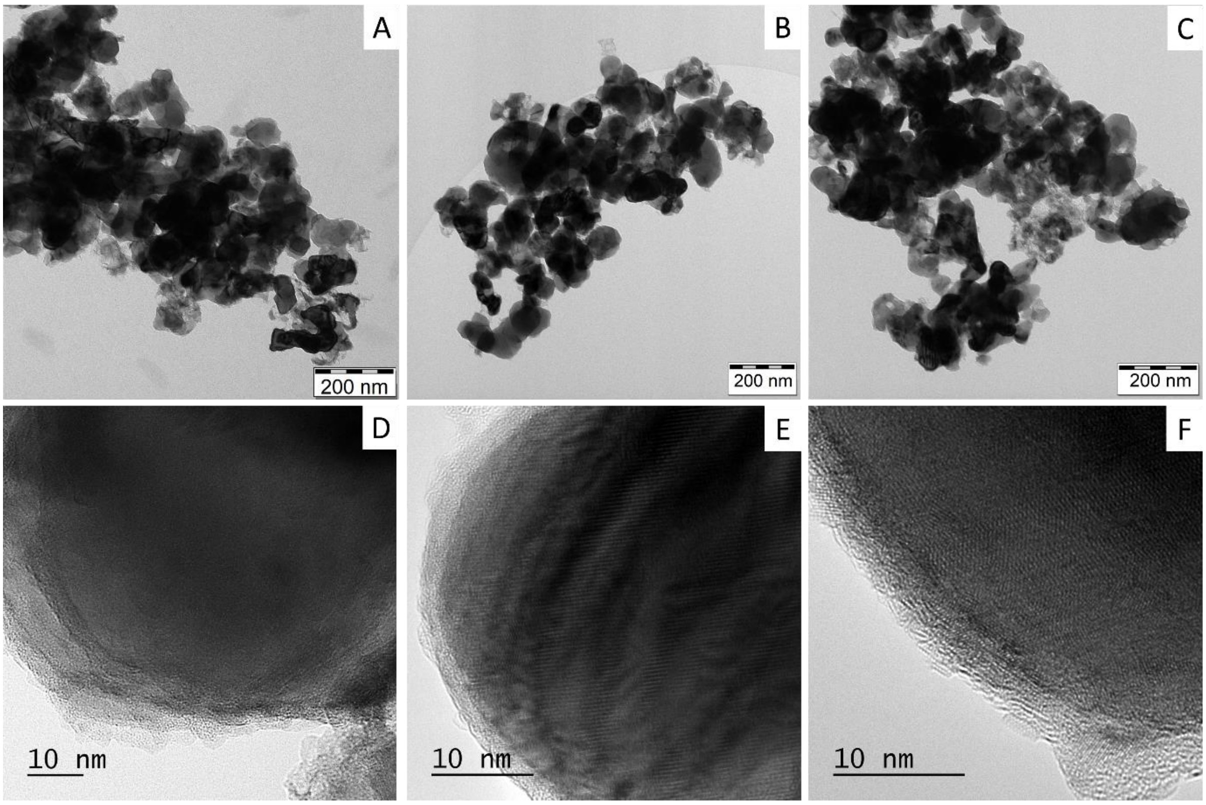

Figure S2. Transmission electron micrographs of fresh particles at two magnifications: (A, D) nZVI, (B, E) γ'-Fe_xN, (C, F) ε-Fe_xN.

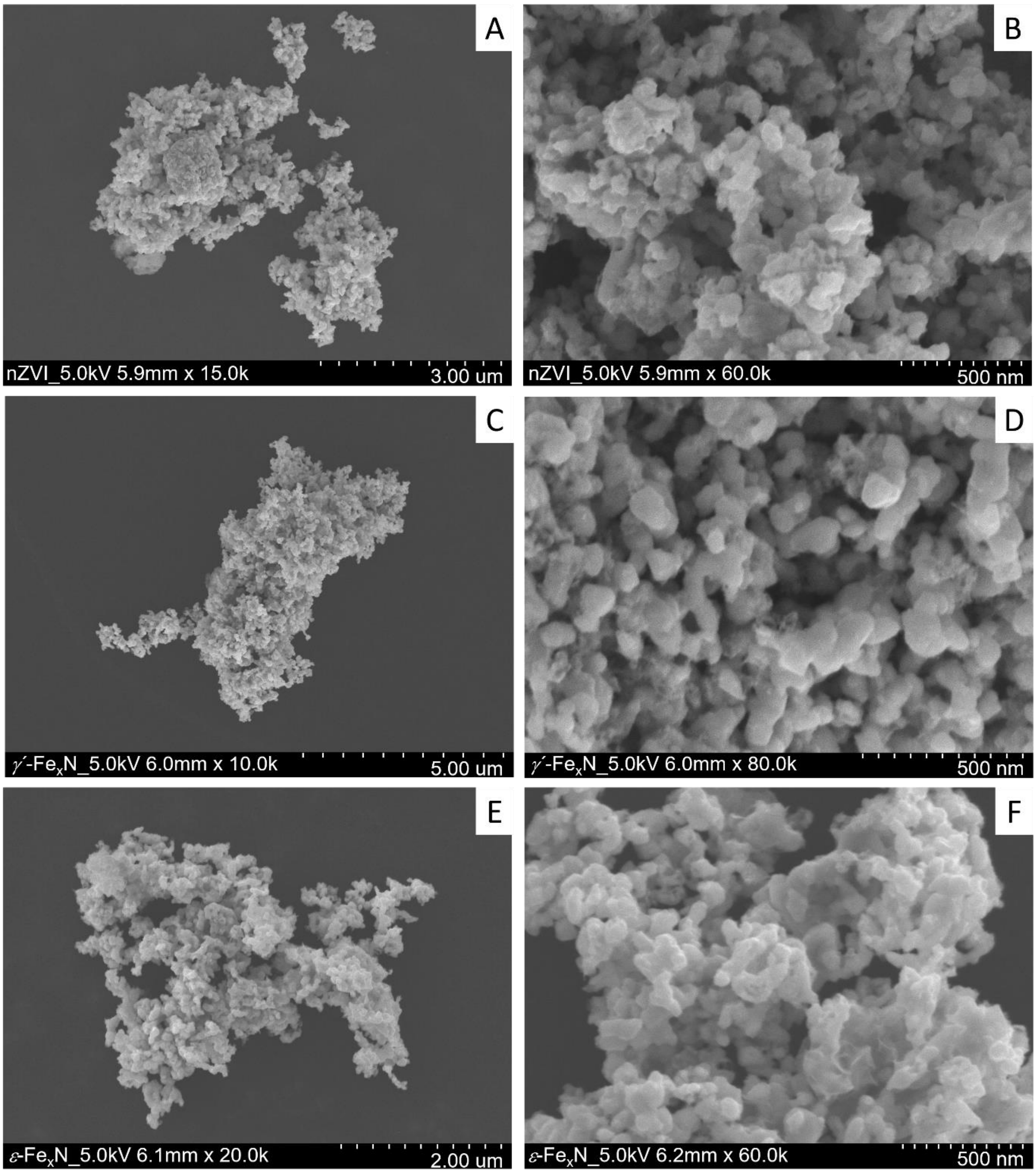

Figure S3. Scanning electron micrographs of fresh particles at two magnifications: (A, B) nZVI, (C, D) γ'-Fe_xN, (E, F) ε-Fe_xN.

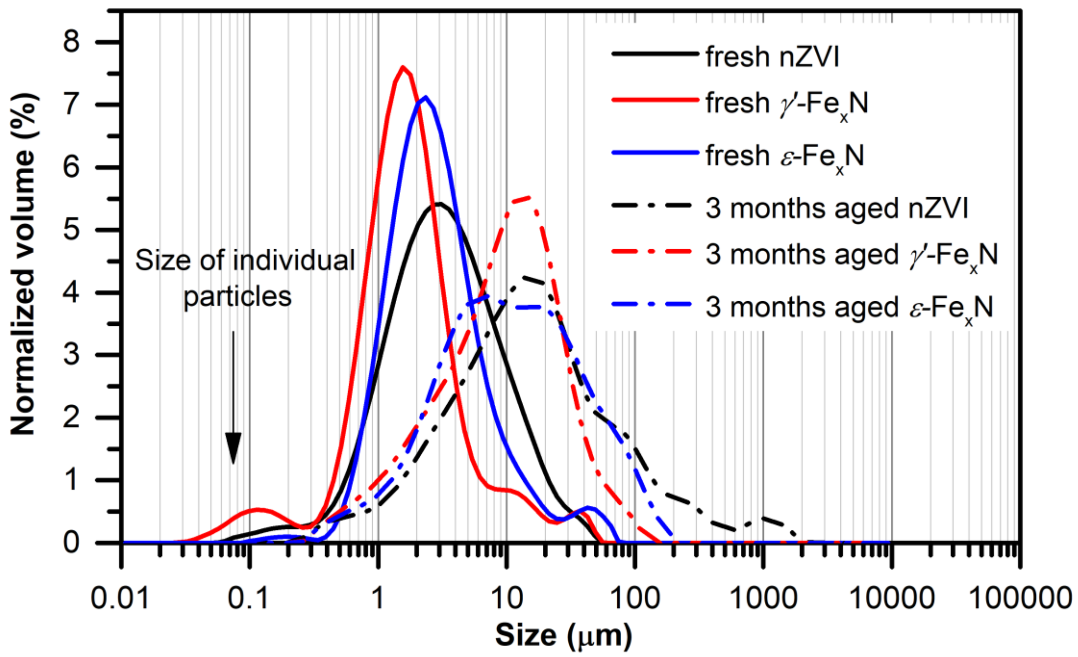

Figure S4. Particle agglomerate size distribution of fresh and three months aged suspensions of Fe_xN and nZVI particles in MHW.

Table S6. Agglomerate size characteristics of fresh and aged particles in suspension with uncertainties expressed as standard deviation (SD).

Particle type	Volume weighted mean (nm)	$d_{0.1}$ (nm)	$d_{0.5}$ / median (nm)	$d_{0.9}$ (nm)
Fresh nZVI	5 210 ± 304	879 ± 6	3 145 ± 283	11 877 ± 720
Fresh γ'-Fe_xN	3 169 ± 80	593 ± 71	1 626 ± 64	5 516 ± 359
Fresh ε-Fe_xN	4 901 ± 33	1 037 ± 121	2 559 ± 272	9 162 ± 1 406
Aged nZVI	61 001 ± 2 437	2 336 ± 26	14 543 ± 266	107 555 ± 6 058
Aged γ'-Fe_xN	14 776 ± 543	1 741 ± 59	10 393 ± 820	32 041 ± 1 065
Aged ε-Fe_xN	21 103 ± 169	2 017 ± 69	10 591 ± 416	54 633 ± 689

Table S7. Size of individual fresh Fe_xN and nZVI particles and thickness of surface (oxyhydr)oxide shell; the uncertainties are expressed as standard deviation (SD). Statistically significant differences ($p < 0.005$) to pristine nZVI are labeled with an *.

Particle type	Mean size ± SD (nm)	Mean shell thickness ± SD (nm)
nZVI	72 ± 26	3.8 ± 1.3
γ'-Fe_xN	73 ± 21	3.2 ± 0.8*
ε-Fe_xN	77 ± 21	3.7 ± 0.7

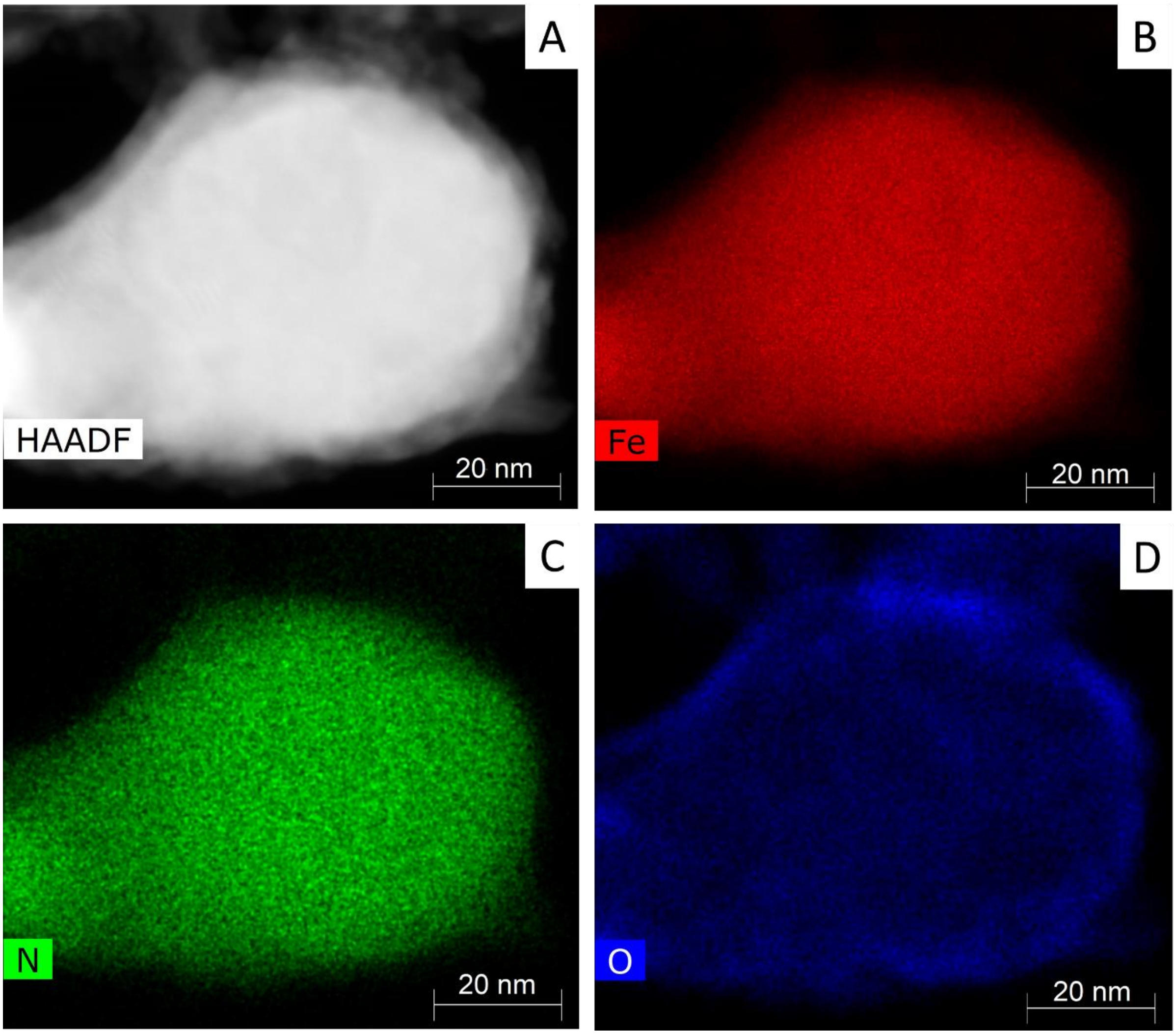

Figure S5. STEM-EDS mappings of a γ'-Fe_xN particle: (A) high-angle annular dark-field (HAADF) image, (B) Fe (Kα) mapping, (C) N (Kα) mapping, (D) O (Kα) mapping.

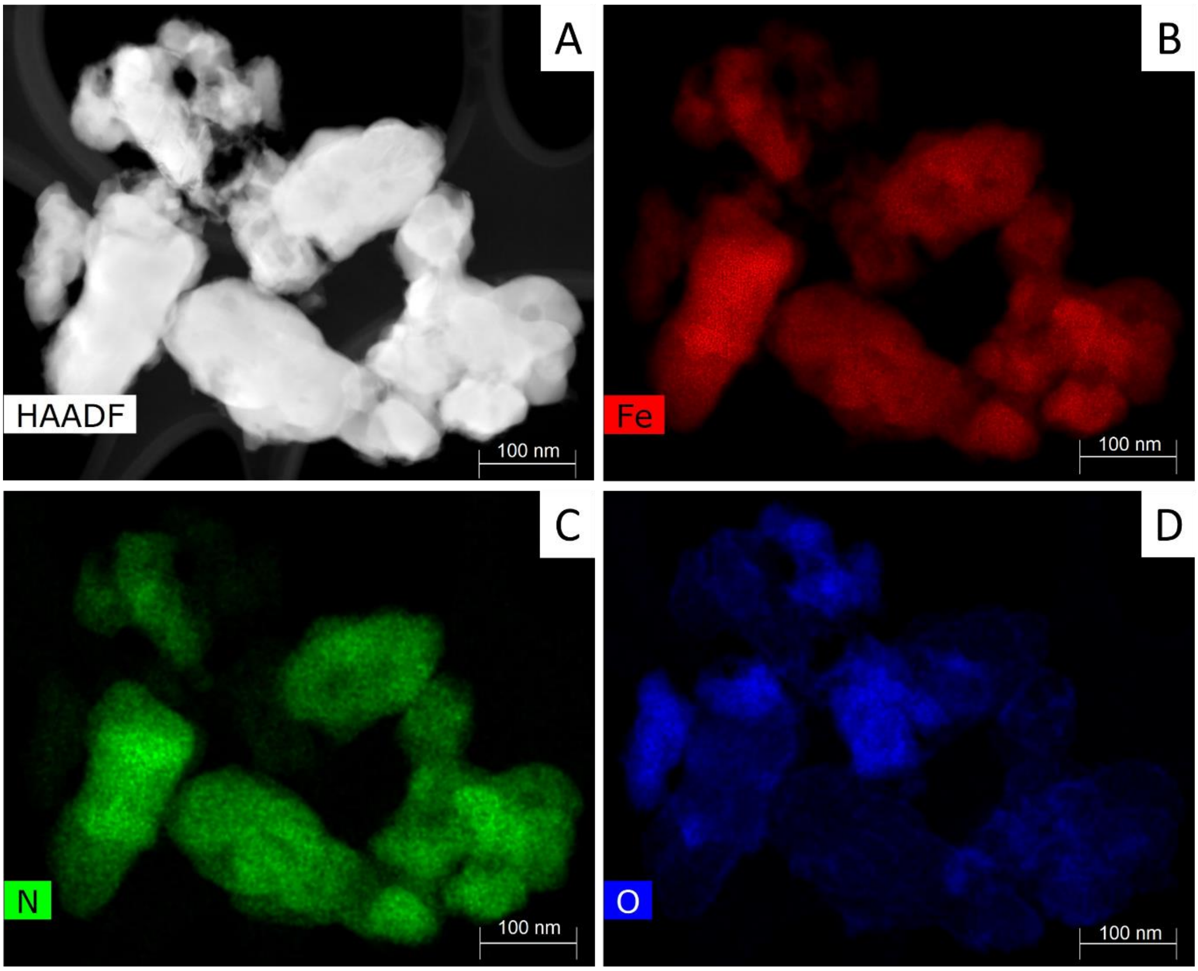

Figure S6. STEM-EDS mappings of ε-Fe_xN particles: (A) high-angle annular dark-field (HAADF) image, (B) Fe (Kα) mapping, (C) N (Kα) mapping, (D) O (Kα) mapping.

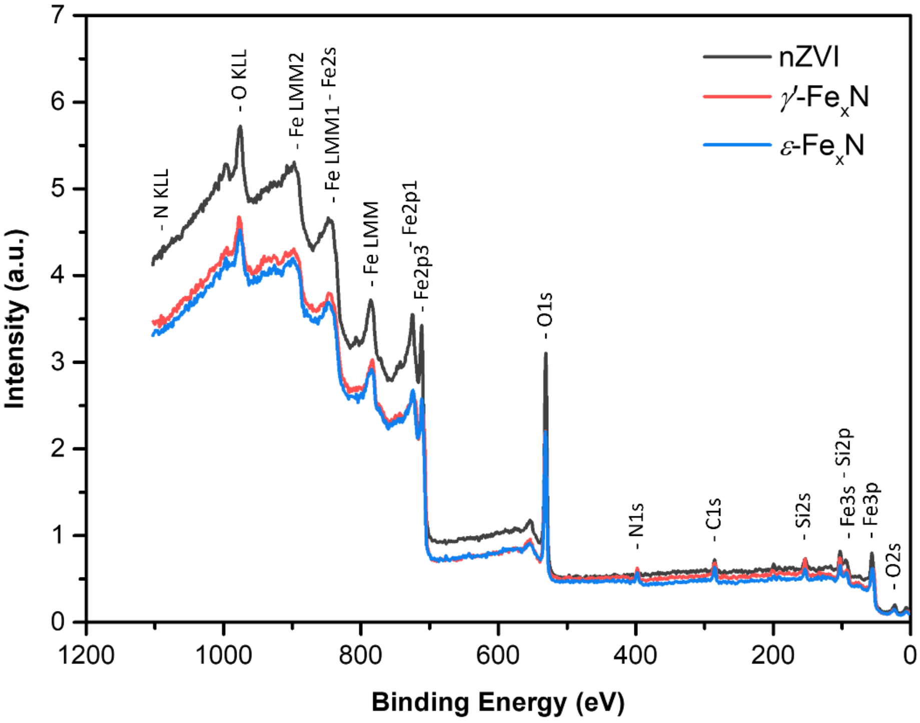

Figure S7. XPS survey spectra of fresh Fe_xN and nZVI particles.

Table S8. Surface element quantification of fresh Fe_xN and nZVI particles by XPS survey scan.

Particle type	Atomic ratio %				
	Fe 2p	O 1s	N 1s	C 1s*	Si 2p†
nZVI	30.3	57.6	0.3	6.4	5.4
γ'-Fe_xN	24.0	51.8	4.3	11.2	8.7
ε-Fe_xN	24.5	50.7	5.5	12.4	6.9

* Carbon signal originates from the sample preparation.

† Silicon was present in the pristine nZVI as an impurity.

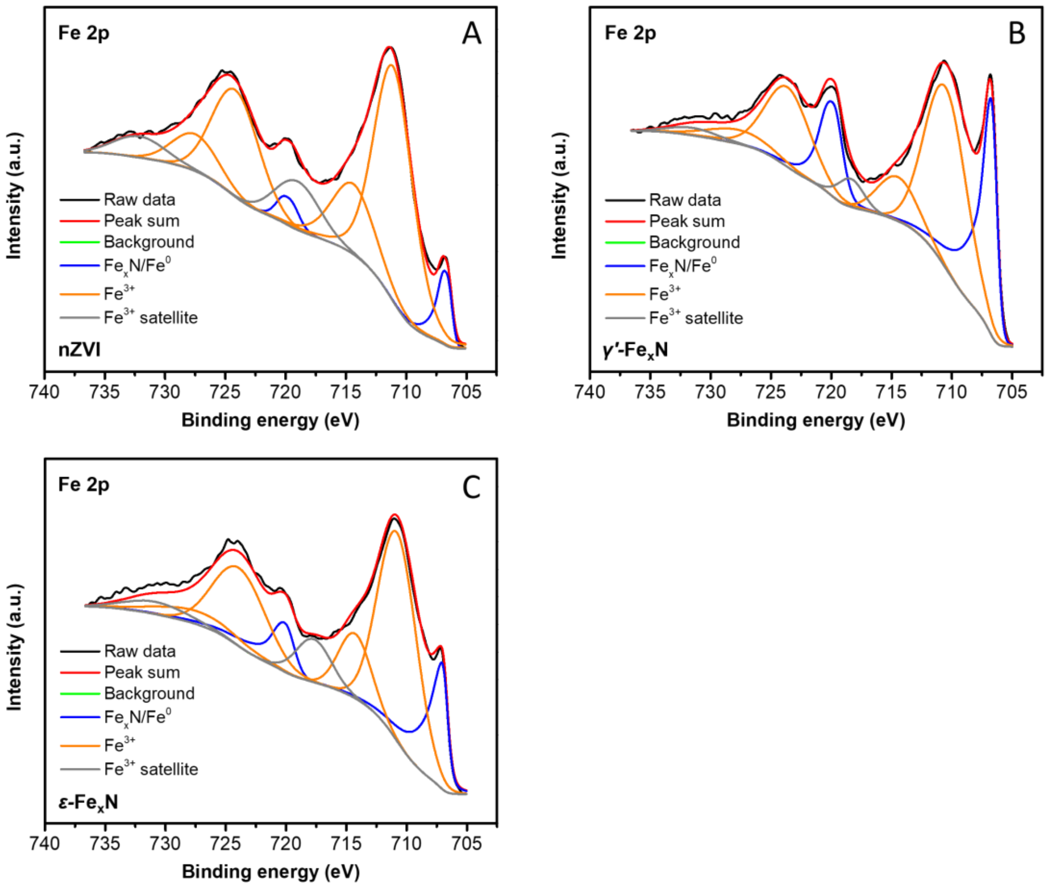

Figure S8. Fe 2p XPS narrow region spectra of fresh (A) nZVI, (B) γ'-Fe_xN, and (C) ε-Fe_xN particles.

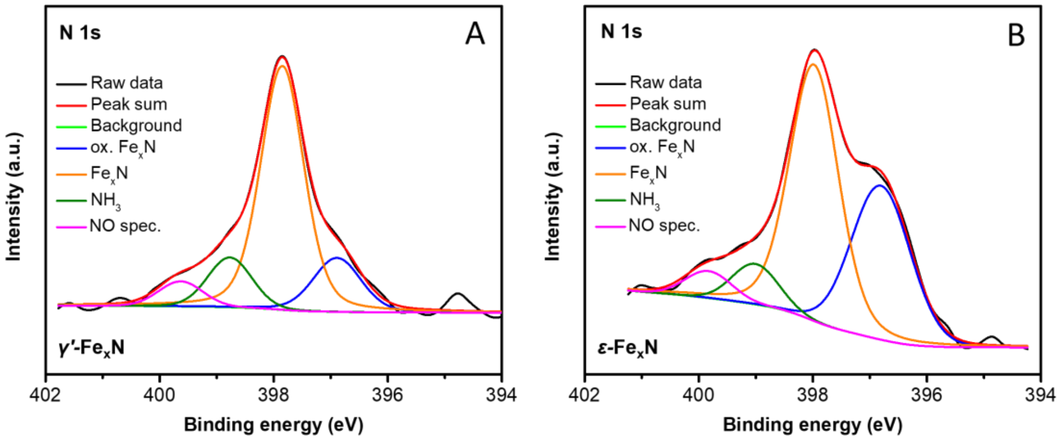

Figure S9. N 1s XPS narrow region spectra of fresh (A) γ'-Fe_xN and (B) ε-Fe_xN particles.

Table S9. Fe 2p and N 1s binding energies (eV) and atomic ratios obtained by XPS for fresh Fe_xN and nZVI particles.

Particle type	Level	State	Binding energies (eV)	Relative area (%)
nZVI	Fe (2p)*	Fe_xN/Fe^0	706.6, 719.7	4.85, 2.42
		Fe^{3+}	711.1, 724.2, 714.4, 727.5	39.63, 19.82, 13.56, 6.78
	N (1s)	Fe_xN	--	--
		ox. Fe_xN	--	--
		NH_3	--	--
		NO species	--	--
γ'-Fe_xN	Fe (2 p)*	Fe_xN/Fe^0	706.7, 719.8	21.68, 10.84
		Fe^{3+}	710.5, 723.6, 714.5, 727.6	33.86, 16.93, 7.89, 3.95
	N (1s)	Fe_xN	397.9	66.48
		ox. Fe_xN	396.9	15.27
		NH_3	398.8	12.54
		NO species	399.6	5.71
ε-Fe_xN	Fe (2 p)*	Fe_xN/Fe^0	707.0, 720.1	13.66, 6.83
		Fe^{3+}	710.8, 723.9, 714.3, 727.4	36.65, 18.32, 9.50, 4.75
	N (1s)	Fe_xN	398.0	48.17
		ox. Fe_xN	396.8	36.36
		NH_3	399.0	8.82
		NO species	399.8	6.65

* Fe^{3+} satellite peaks are not shown in the quantification.

Table S10. BET specific surface area of investigated nanoparticles with uncertainties expressed as standard deviation (SD).

Particle type	BET SSA ($m^2 g^{-1}$)
nZVI	22.7 ± 1.0
γ'-Fe_xN	18.9 ± 0.3
ε-Fe_xN	23.2 ± 0.6

Table S11. Elemental composition of investigated nanoparticles with uncertainties expressed as standard deviation (SD).

**Particle type**	**Fe_{tot} content (wt. %)**	**Reducing capacity (recalculated to Fe^0 wt. %)**	**N_{tot} content (wt. %)***	**N_{tot} content (wt. %)†**	**C content (wt. %)***
nZVI	99.3 ± 0.4	83.4 ± 1.0	n.d.‡	n.d.‡	0.32 ± 0.02
γ'-Fe_xN	96.2 ± 1.1	55.7 ± 0.5	5.27 ± 0.03	5.30 ± 0.06	0.31 ± 0.02
ε-Fe_xN	91.2 ± 2.3	20.9 ± 0.7	7.51 ± 0.08	7.74 ± 0.04	0.32 ± 0.01

* Determined by elemental analysis.

† Determined from the sum of concentrations of dissolved nitrogen species ($NH_{3(aq)}$, NO_2^-, and NO_3^-) after acid digestion.

‡ Not detected.

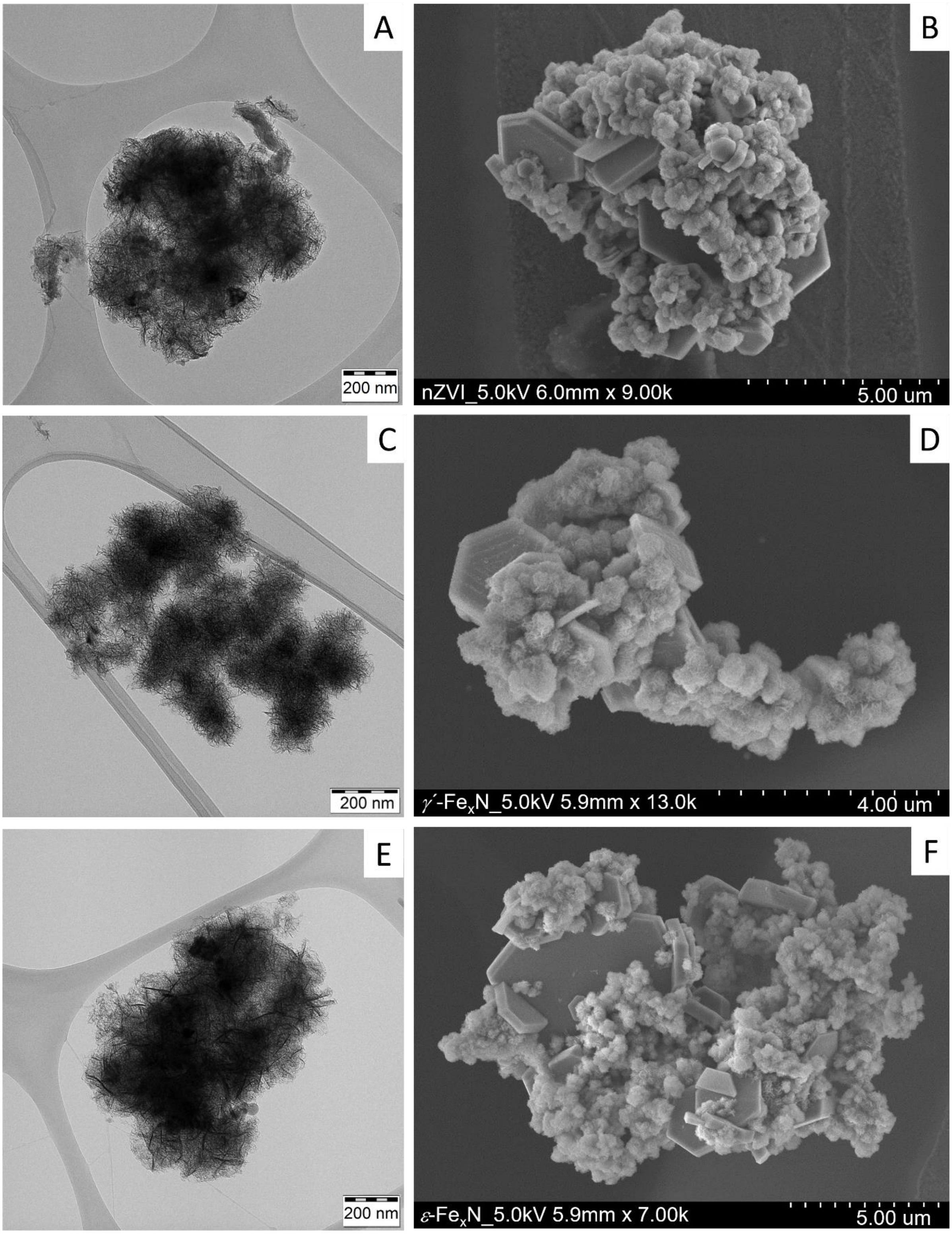

Figure S10. Transmission and scanning electron micrographs of particles aged three months in MHW: (A, B) nZVI, (C, D) γ'-Fe_xN, (E, F) ε-Fe_xN.

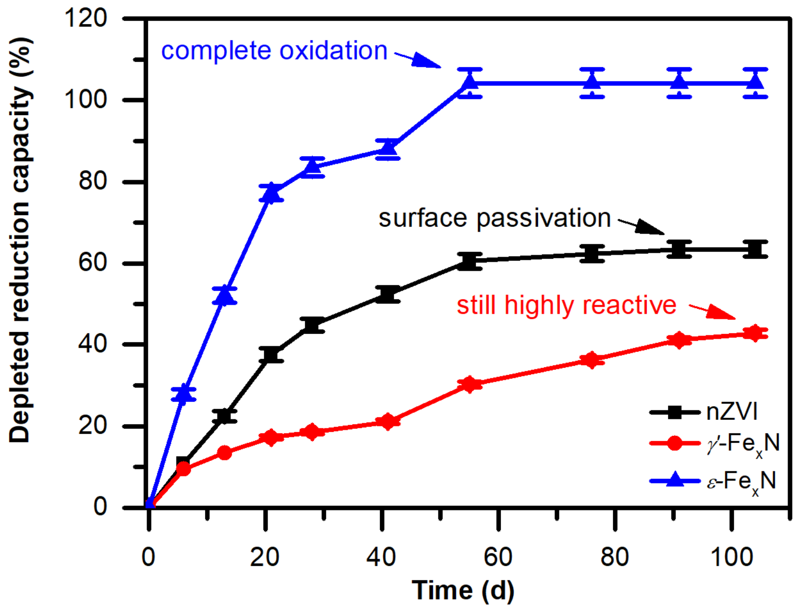

Figure S11. Depletion of particle reducing capacity over the course of aging calculated from the volume of hydrogen evolved during the aging experiment and the volume of hydrogen evolved when fresh particles were digested with HCl.

Table S12. Concentrations of leached ammonia ($NH_{3(aq)}$), nitrite (NO_2^-), and nitrate (NO_3^-) from Fe_xN and nZVI particles in MHW after 3 months of aging.

**Particle type**	**Concentration ± SD (mg L^{-1})**		
	**$NH_{3(aq)}$**	**NO_2^-**	**NO_3^-**
nZVI	< 0.78	< 1.52	< 5.65
γ'-Fe_xN	35.8 ± 1.1	< 1.52	< 5.65
ε-Fe_xN	79.5 ± 1.2	< 1.52	< 5.65

Table S13. TCE dechlorination rates, hydrogen evolution rates, and estimated longevity of Fe_xN and pristine nZVI particles.

Particle type		TCE removal, k_{obs} (10^{-3} hr^{-1})	TCE removal, k_{SA} (10^{-3} L m^{-2} hr^{-1})	Initial H_2 evolution rate*, k_m (μmol g^{-1} hr^{-1})	Initial H_2 evolution rate*, k_{SA} (μmol m^{-2} d^{-1})	Estimated longevity† (days)
Fresh	nZVI	3.81 ± 0.10	0.229 ± 0.011	23.9 ± 0.4	25.2 ± 1.1	35.1
	γ'-Fe_xN	74.6 ± 1.2	5.39 ± 0.12	6.45 ± 0.22	8.19 ± 0.31	104.2
	ε-Fe_xN	20.4 ± 0.4	1.20 ± 0.04	8.60 ± 0.32	8.90 ± 0.41	31.8
Aged 3 months	nZVI	1.00 ± 0.06	--	--	--	--
	γ'-Fe_xN	28.0 ± 0.7	--	--	--	--
	ε-Fe_xN	1.02 ± 0.12	--	--	--	--

* Calculated from the linear portion of the hydrogen evolution curve (t < 9 days).

† Calculated from the amount of hydrogen evolved over three weeks of reaction.

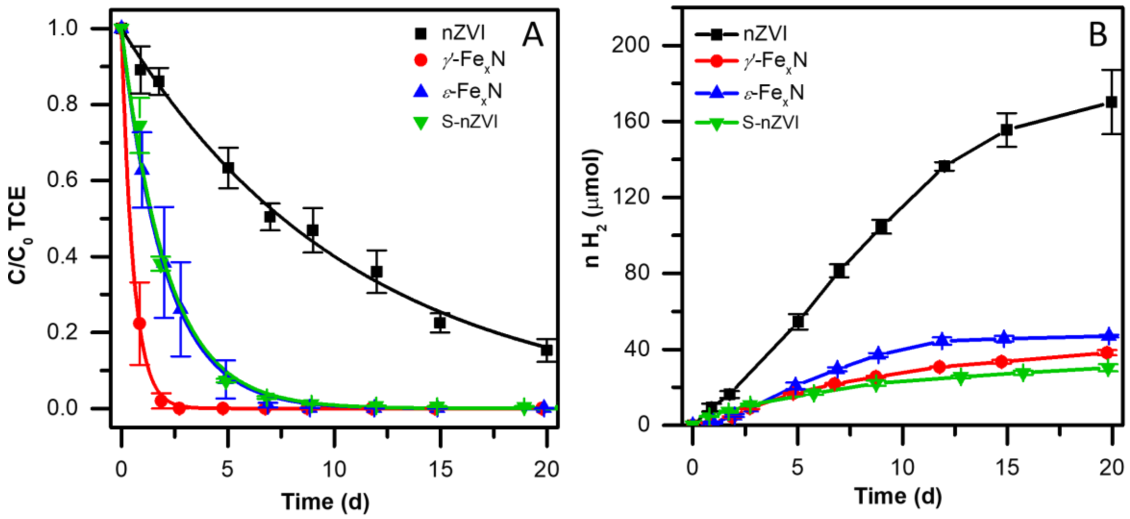

Figure S12. (A) TCE removal by fresh nZVI, Fe_xN, and S-nZVI particles (S/Fe mass ratio 0.94/100); (B) corresponding hydrogen evolution during the TCE degradation experiment. The reactions were carried out at an initial TCE concentration of 20 mg L^{-1} and particle concentration of 1 g L^{-1}. S-nZVI was synthesized according to our previous study.[8] Whiskers indicate standard deviation (SD).

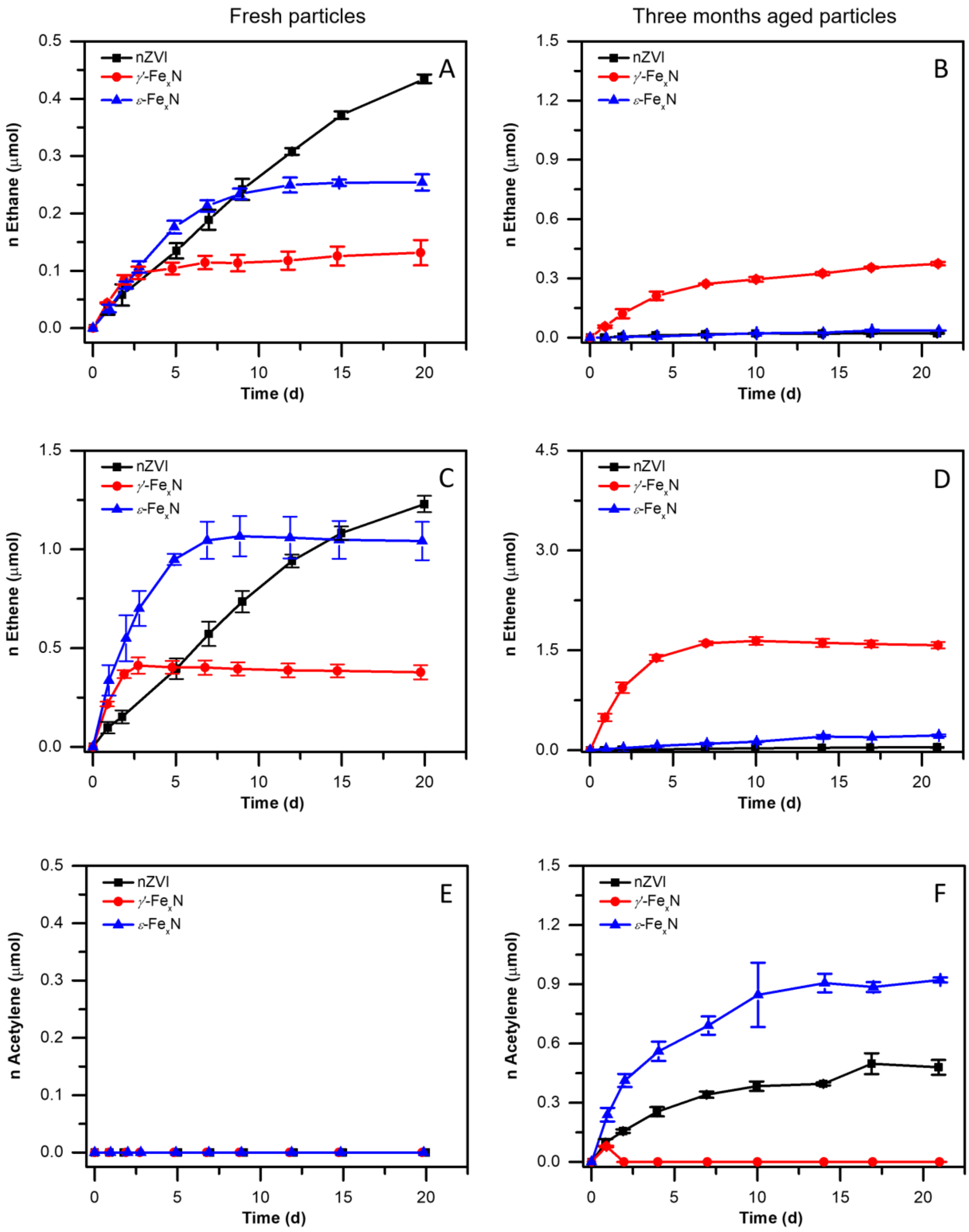

Figure S13. Kinetics of formation of C_2-hydrocarbons ethane (A, B), ethene (C, D), and acetylene (E, F) during TCE dechlorination experiments with fresh and three months aged particles, respectively. The y-axis range for aged particles is three times bigger since the volume of the reaction mixture was three times greater than for experiments with fresh particles. Whiskers indicate standard deviation (SD).

Table S14. Tentatively identified products at the end of the TCE dechlorination experiment with fresh γ'-Fe_xN nanoparticles using non-target screening. Compound identification was performed in the Agilent MassHunter Unknown Analysis software. Only compounds with a match factor > 90 with the NIST 17 mass spectral library are shown. The non-target analysis was performed with four replicates.

**Compound**	**Match factors**
3-methylpent-2-ene	94.5, 94.3, 94.2, 93.8
1-methylcyclopentene	94, 91.7, 91.1, 90.9
1,2-dimethylcyclopropane	93.7, 92.8, 92.8, 91.4
Methylcyclopentane	91.1, 91.1
2-methylbut-2-ene	92.6
Pent-2-ene	92.3
2-methylpent-2-ene	92.2
Hex-2-ene	90.9
2,5,5-trimethylcyclopentadiene	90.3
2,3-dimethylbut-1-ene	90.1

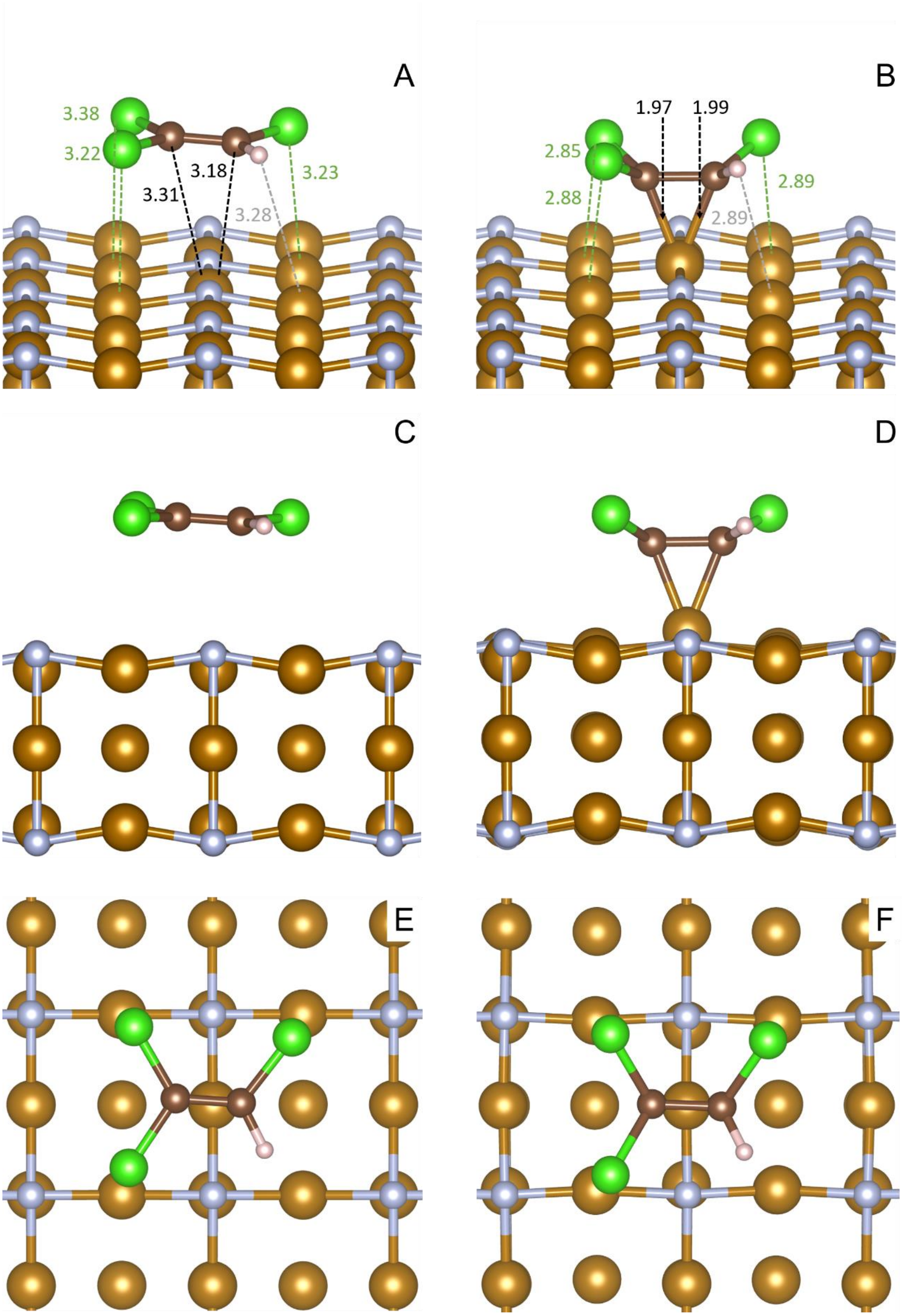

Figure S14. Physisorbed (left) and chemisorbed (right) configurations of TCE on the γ'-$Fe_4N(001)$ surface: (A, B) overall view with selected interatomic distances depicted in Å; (C, D) side view; (E, F) top view. Visualizations were prepared using VESTA 3.[35]

Table S15. The geometry of TCE in the gas phase, in the physisorbed state, and the chemisorbed state on the γ'-$Fe_4N(001)$ surface.

Parameter	Gas-phase	Physisorbed state	Chemisorbed state
C=C bond length (Å)	1.343	1.348	1.442
C–Cl1* bond length (Å)	1.721	1.718	1.791
C–Cl2* bond length (Å)	1.708	1.707	1.770
C–Cl3* bond length (Å)	1.707	1.706	1.766
C–H bond length (Å)	1.089	1.089	1.093
Cl1–C–C–Cl3* dihedral angle (°)	180.0	177.0	136.8
Cl2–C–C–H* dihedral angle (°)	180.0	179.6	139.2

* Atom positions:

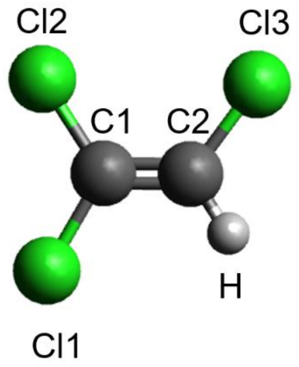

Table S16. DFT-calculated homolytic bond dissociation energies (BDE) of C–Cl bonds in the isolated TCE molecule in the gas phase (VASP and TURBOMOLE) and in the solvent (COSMO model as implemented in TURBOMOLE). Energies are in kJ mol^{-1}.

C–Cl bond position	BDE (VASP)	BDE (TURBOMOLE)	BDE (COSMO)
C–Cl1*	395.5	379.5	376.2
C–Cl2*	405.1	388.7	384.8
C–Cl3*	425.6	410.5	406.4

* For chlorine atom positions please refer to Table S15.

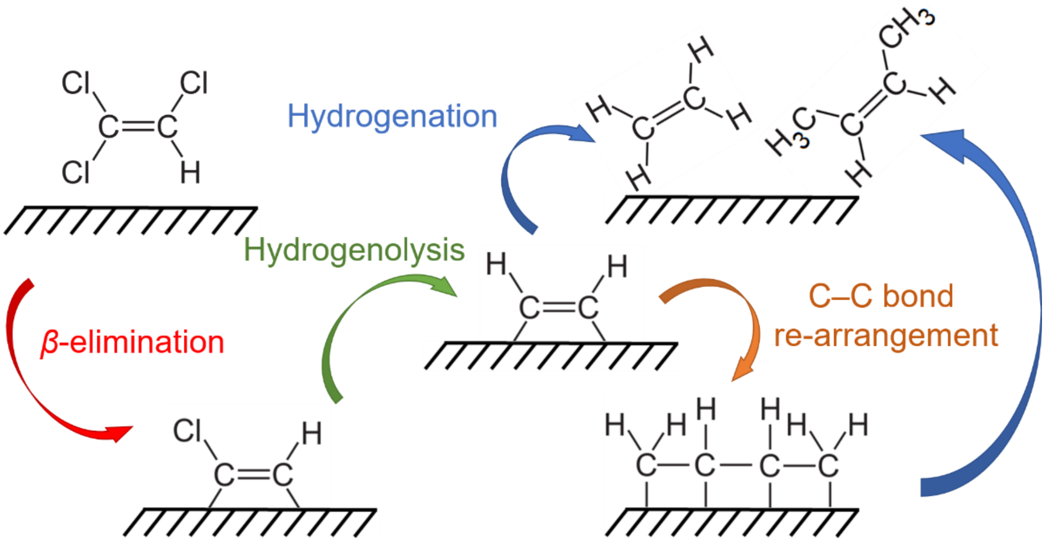

Figure S15. Proposed multi-step reaction mechanism of TCE dechlorination by Fe_xN particles.

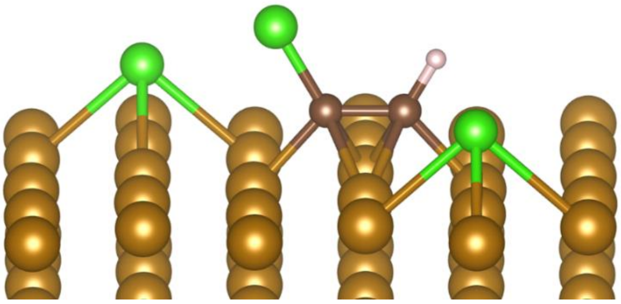

Figure S16. Configuration of adsorbed TCE on the *α*-Fe(110) surface resulting from the full geometry optimization (TCE molecule initially placed ca. 5 Å above the Fe slab).

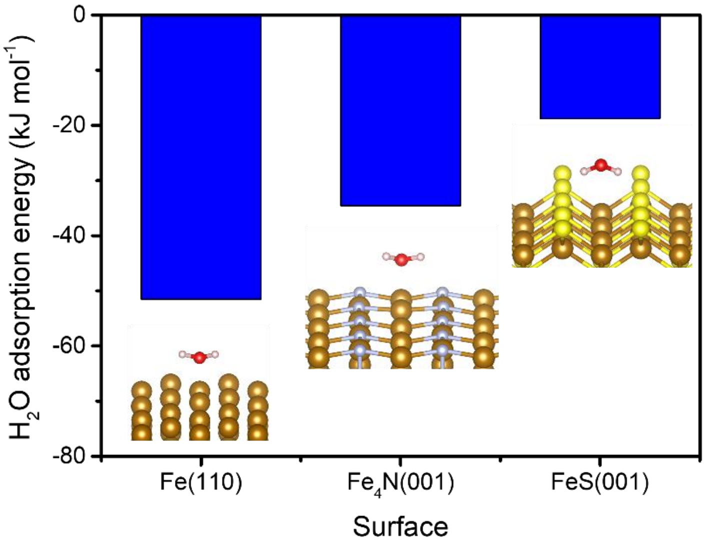

Figure S17. PBE-D3 calculated water adsorption energies and configurations on the α-Fe(110), γ'-Fe_4N(001), and FeS(001) surfaces.

References

(1) US EPA. *Methods for Measuring the Acute Toxicity of Effluents and Receiving Waters to Freshwater and Marine Organisms, Fifth Edition*; Washington, DC, 2002.

(2) Appelo, C. A. J.; Postma, D. *Geochemistry, Groundwater and Pollution*, 2nd ed.; A.A. Balkema Publishers: Amsterdam, 2005.

(3) PONNAMPERUMA, F. N.; TIANCO, E. M.; LOY, T. A. IONIC STRENGTHS OF THE SOLUTIONS OF FLOODED SOILS AND OTHER NATURAL AQUEOUS SOLUTIONS FROM SPECIFIC CONDUCTANCE. *Soil Sci.* **1966**, *102* (6), 408–413. https://doi.org/10.1097/00010694-196612000-00009.

(4) Rietveld, H. M. A Profile Refinement Method for Nuclear and Magnetic Structures. *J. Appl. Crystallogr.* **1969**, *2* (2), 65–71. https://doi.org/10.1107/S0021889869006558.

(5) Pechousek, J.; Prochazka, R.; Jancik, D.; Frydrych, J.; Mashlan, M. Universal LabVIEW-Powered Mössbauer Spectrometer Based on USB, PCI or PXI DevicesI. In *International Conference on the Applications of the Mössbauer Effect (ICAME 2009)*; Muller, H., Reissner, M., Steiner, W., Wiesinger, G., Eds.; IOP Publishing Ltd: Bristol, 2010.

(6) Pechousek, J.; Jancik, D.; Frydrych, J.; Navarik, J. N.; Novak, P. Setup of Mossbauer Spectrometers at RCPTM. In *Mossbauer Spectroscopy in Materials Science - 2012*; American Institute of Physics: Melville, New York, 2013; pp 186–194.

(7) Klencsár, Z.; Kuzmann, E.; Vértes, A. User-Friendly Software for Mössbauer Spectrum Analysis. *J. Radioanal. Nucl. Chem. Artic.* **1996**, *210* (1), 105–118. https://doi.org/10.1007/BF02055410.

(8) Brumovský, M.; Filip, J.; Malina, O.; Oborná, J.; Sracek, O.; Reichenauer, T. G.; Andrýsková, P.; Zbořil, R. Core–Shell Fe/FeS Nanoparticles with Controlled Shell Thickness for Enhanced Trichloroethylene Removal. *ACS Appl. Mater. Interfaces* **2020**, *12* (31), 35424–35434. https://doi.org/10.1021/acsami.0c08626.

(9) Schöftner, P.; Waldner, G.; Lottermoser, W.; Stöger-Pollach, M.; Freitag, P.; Reichenauer, T. G. Electron Efficiency of NZVI Does Not Change with Variation of Environmental Parameters. *Sci. Total Environ.* **2015**, *535*, 69–78. https://doi.org/10.1016/j.scitotenv.2015.05.033.

(10) Wu, S.; Salcedo, J.; Tang, N.; Waddell, K.; Grimm, R.; German, J. B.; Lebrilla, C. B. Employment of Tandem Mass Spectrometry for the Accurate and Specific Identification of Oligosaccharide Structures. *Anal. Chem.* **2012**, *84* (17), 7456–7462. https://doi.org/10.1021/ac301398h.

(11) Kresse, G.; Hafner, J. Ab Initio Molecular Dynamics for Open-Shell Transition Metals. *Phys. Rev. B* **1993**, *48* (17), 13115–13118. https://doi.org/10.1103/PhysRevB.48.13115.

(12) Kresse, G.; Hafner, J. Ab Initio Molecular-Dynamics Simulation of the Liquid-Metal–Amorphous-Semiconductor Transition in Germanium. *Phys. Rev. B* **1994**, *49* (20), 14251–14269. https://doi.org/10.1103/PhysRevB.49.14251.

(13) Kresse, G.; Furthmüller, J. Efficient Iterative Schemes for Ab Initio Total-Energy Calculations Using a Plane-Wave Basis Set. *Phys. Rev. B* **1996**, *54* (16), 11169–11186. https://doi.org/10.1103/PhysRevB.54.11169.

(14) Kresse, G.; Furthmüller, J. Efficiency of Ab-Initio Total Energy Calculations for Metals and Semiconductors Using a Plane-Wave Basis Set. *Comput. Mater. Sci.* **1996**, *6* (1), 15–50. https://doi.org/10.1016/0927-0256(96)00008-0.

(15) Blöchl, P. E. Projector Augmented-Wave Method. *Phys. Rev. B* **1994**, *50* (24), 17953–17979. https://doi.org/10.1103/PhysRevB.50.17953.

(16) Kresse, G.; Joubert, D. From Ultrasoft Pseudopotentials to the Projector Augmented-Wave Method. *Phys. Rev. B* **1999**, *59* (3), 1758–1775. https://doi.org/10.1103/PhysRevB.59.1758.

(17) Perdew, J. P.; Burke, K.; Ernzerhof, M. Generalized Gradient Approximation Made Simple. *Phys. Rev. Lett.* **1996**, *77* (18), 3865–3868. https://doi.org/10.1103/PhysRevLett.77.3865.

(18) Grimme, S.; Ehrlich, S.; Goerigk, L. Effect of the Damping Function in Dispersion Corrected Density Functional Theory. *J. Comput. Chem.* **2011**, *32* (7), 1456–1465. https://doi.org/10.1002/jcc.21759.

(19) Monkhorst, H. J.; Pack, J. D. Special Points for Brillouin-Zone Integrations. *Phys. Rev. B* **1976**, *13* (12), 5188–5192. https://doi.org/10.1103/PhysRevB.13.5188.

(20) Shi, Y. J.; Du, Y. L.; Chen, G. Ab Initio Study of Structural and Magnetic Properties of Cubic Fe4N(001) Surface. *Solid State Commun.* **2012**, *152* (16), 1581–1584. https://doi.org/10.1016/j.ssc.2012.05.017.

(21) Shirane, G.; Takei, W. J.; Ruby, S. L. Mössbauer Study of Hyperfine Fields and Isomer Shifts in Fe4N and (Fe, Ni)4N. *Phys. Rev.* **1962**, *126* (1), 49–52. https://doi.org/10.1103/PhysRev.126.49.

(22) Kuhnen, C. A.; de Figueiredo, R. S.; Drago, V.; da Silva, E. Z. Mössbauer Studies and Electronic Structure of Γ′-Fe4N. *J. Magn. Magn. Mater.* **1992**, *111* (1–2), 95–104. https://doi.org/10.1016/0304-8853(92)91062-X.

(23) Frazer, B. C. Magnetic Structure of Fe4N. *Phys. Rev.* **1958**, *112* (3), 751–754. https://doi.org/10.1103/PhysRev.112.751.

(24) Jacobs, H.; Rechenbach, D.; Zachwieja, U. Structure Determination of Γ′-Fe4N and ϵ-Fe3N. *J. Alloys Compd.* **1995**, *227* (1), 10–17. https://doi.org/10.1016/0925-8388(95)01610-4.

(25) Błoński, P.; Kiejna, A. Structural, Electronic, and Magnetic Properties of Bcc Iron Surfaces. *Surf. Sci.* **2007**, *601* (1), 123–133. https://doi.org/10.1016/j.susc.2006.09.013.

(26) Owen, E. A.; Williams, G. I. A Low-Temperature X-Ray Camera. *J. Sci. Instrum.* **1954**, *31* (2), 49–54. https://doi.org/10.1088/0950-7671/31/2/305.

(27) Ayuela, A.; March, N. H. The Magnetic Moments and Their Long-Range Ordering for Fe Atoms in a Wide Variety of Metallic Environments. *Int. J. Quantum Chem.* **2010**, *110* (15), 2725–2733. https://doi.org/10.1002/qua.22764.

(28) Kolos, M.; Tunega, D.; Karlický, F. A Theoretical Study of Adsorption on Iron Sulfides towards Nanoparticle Modeling. *Phys. Chem. Chem. Phys.* **2020**, *22* (40), 23258–23267. https://doi.org/10.1039/D0CP02988B.

(29) Bučko, T.; Hafner, J.; Ángyán, J. G. Geometry Optimization of Periodic Systems Using Internal Coordinates. *J. Chem. Phys.* **2005**, *122* (12), 124508. https://doi.org/10.1063/1.1864932.

(30) Letchworth-Weaver, K.; Arias, T. A. Joint Density Functional Theory of the Electrode-Electrolyte Interface: Application to Fixed Electrode Potentials, Interfacial Capacitances, and Potentials of Zero Charge. *Phys. Rev. B* **2012**, *86* (7), 075140. https://doi.org/10.1103/PhysRevB.86.075140.

(31) Mathew, K.; Sundararaman, R.; Letchworth-Weaver, K.; Arias, T. A.; Hennig, R. G. Implicit

Solvation Model for Density-Functional Study of Nanocrystal Surfaces and Reaction Pathways. *J. Chem. Phys.* **2014**, *140* (8), 084106. https://doi.org/10.1063/1.4865107.

(32) Mathew, K.; Kolluru, V. S. C.; Mula, S.; Steinmann, S. N.; Hennig, R. G. Implicit Self-Consistent Electrolyte Model in Plane-Wave Density-Functional Theory. *J. Chem. Phys.* **2019**, *151* (23), 234101. https://doi.org/10.1063/1.5132354.

(33) Tomasi, J.; Mennucci, B.; Cammi, R. Quantum Mechanical Continuum Solvation Models. *Chem. Rev.* **2005**, *105* (8), 2999–3094. https://doi.org/10.1021/cr9904009.

(34) Henkelman, G.; Uberuaga, B. P.; Jónsson, H. A Climbing Image Nudged Elastic Band Method for Finding Saddle Points and Minimum Energy Paths. *J. Chem. Phys.* **2000**, *113* (22), 9901–9904. https://doi.org/10.1063/1.1329672.

(35) Momma, K.; Izumi, F. VESTA 3 for Three-Dimensional Visualization of Crystal, Volumetric and Morphology Data. *J. Appl. Crystallogr.* **2011**, *44* (6), 1272–1276. https://doi.org/10.1107/S0021889811038970.

(36) Ahlrichs, R.; Bär, M.; Häser, M.; Horn, H.; Kölmel, C. Electronic Structure Calculations on Workstation Computers: The Program System Turbomole. *Chem. Phys. Lett.* **1989**, *162* (3), 165–169. https://doi.org/10.1016/0009-2614(89)85118-8.

(37) Weigend, F. Accurate Coulomb-Fitting Basis Sets for H to Rn. *Phys. Chem. Chem. Phys.* **2006**, *8* (9), 1057. https://doi.org/10.1039/b515623h.

(38) Weigend, F.; Ahlrichs, R. Balanced Basis Sets of Split Valence, Triple Zeta Valence and Quadruple Zeta Valence Quality for H to Rn: Design and Assessment of Accuracy. *Phys. Chem. Chem. Phys.* **2005**, *7* (18), 3297. https://doi.org/10.1039/b508541a.

(39) Klamt, A.; Schüürmann, G. COSMO: A New Approach to Dielectric Screening in Solvents with Explicit Expressions for the Screening Energy and Its Gradient. *J. Chem. Soc., Perkin Trans. 2* **1993**, No. 5, 799–805. https://doi.org/10.1039/P29930000799.
